# Metabolic Reprogramming in Glioblastoma Stem Cells Promotes Radiation Resistance Through a H3K18la/USP30/MBOAT2 Axis

**DOI:** 10.1002/advs.77044

**Published:** 2026-08-12

**Authors:** Zong Miao, Wei Gu, Yimin Ren, Chenfei Lu, Wanzhi Cai, Junnan Lu, Zhongyuan Bao, Yanan Zhou, Rong Li, Ke Jin, Chao Chen, Hongxiang Wang, Lei Xu, Juxiang Chen

**Affiliations:** ^1^ Department of Neurosurgery Changhai Hospital Naval Medical University (Second Military Medical University) Shanghai China; ^2^ Department of Neurosurgery The First Affiliated Hospital of Nanjing Medical University Nanjing China; ^3^ Department of Hematology Huashan Hospital Fudan University Shanghai China; ^4^ Neurovascular Center Changhai Hospital Naval Medical University (Second Military Medical University) Shanghai China

**Keywords:** ferroptosis, glioblastoma, lactylation, MBOAT2, ubiquitination

## Abstract

Mesenchymal glioma stem cells (MES GSCs) are closely associated with glioblastoma radioresistance, yet the mechanisms linking MES‐state maintenance to ferroptosis resistance remain incompletely defined. Here, we show that MES GSCs exhibit enhanced glycolytic activity and lactate production, driven in part by MES‐associated transcriptional regulators that promote LDHA expression. LDHA‐derived lactate induces p300‐dependent H3K18 lactylation, which enhances USP30 transcription. USP30 subsequently stabilizes the lipid‐remodeling enzyme MBOAT2 by limiting its ubiquitination, leading to phosphatidylethanolamine remodeling toward ferroptosis‐resistant PE‐MUFA species. Disruption of this LDHA–H3K18la–USP30–MBOAT2 axis by LDHA inhibition, impaired H3K18 lactylation, USP30 inhibition, or MBOAT2 depletion increases lipid peroxidation, promotes ferroptosis, and sensitizes MES GSCs to irradiation. Lipid rescue experiments further identify PE‐MUFA remodeling as a functional mediator of MBOAT2‐dependent ferroptosis resistance. In intracranial xenografts, combined targeting of glycolysis and USP30 enhances radiotherapeutic efficacy without obvious treatment‐associated body weight loss under the tested conditions. These findings reveal a metabolic–epigenetic–lipid remodeling circuit that protects MES GSCs from ferroptosis and promotes radioresistance.

## Graphical abstract

LDHA‐driven lactate production in mesenchymal glioma stem cells promotes p300‐dependent H3K18 lactylation, activates USP30 transcription, and stabilizes MBOAT2 to remodel phosphatidylethanolamines toward ferroptosis‐resistant PE‐MUFA species. Disrupting this metabolic–epigenetic–lipid circuit enhances ferroptosis and sensitizes glioblastoma to radiotherapy.

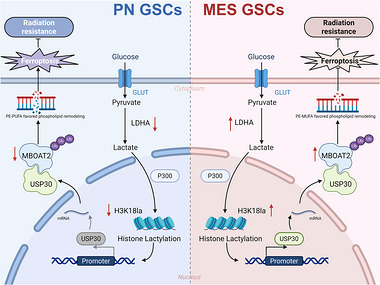

## 1 Introduction

Glioblastoma (GBM) is the most aggressive primary malignancy of the central nervous system and remains largely incurable despite maximal surgical resection followed by radiotherapy and temozolomide‐based chemotherapy [1, 2]. A major barrier to durable therapeutic response is the persistence of glioma stem cells (GSCs), a self‐renewing tumor cell population that drives tumor propagation, recurrence, and resistance to conventional therapies [3, 4, 5]. Among transcriptionally defined GSC states, mesenchymal (MES) GSCs are particularly associated with aggressive tumor behavior, therapeutic resistance, and poor clinical outcome. MES GSCs display enhanced plasticity, inflammatory signaling, and stress‐adaptive phenotypes, which collectively contribute to radioresistance. However, the metabolic and epigenetic mechanisms that sustain MES characteristics and protect GSCs from radiotherapy‐induced damage remain insufficiently defined.

Ferroptosis, an iron‐dependent form of regulated cell death characterized by lethal lipid peroxidation, has emerged as a significant mechanism in glioma biology and therapy [6, 7]. Distinct from apoptosis, ferroptosis is both morphologically and biochemically unique, presenting a promising strategy to circumvent the inherent apoptosis resistance that hinders conventional glioma treatments [8, 9]. Recent research has underscored that targeting ferroptosis‐associated proteins or utilizing ferroptosis inducers can effectively mitigate therapeutic resistance in GBM [4, 10, 11]. In GBM, ferroptosis sensitivity is influenced by redox homeostasis, cystine uptake through system Xc^−^, glutathione metabolism, GPX4 activity, mitochondrial metabolism, and membrane phospholipid composition [12, 13]. Pharmacological inhibition of GPX4 or system Xc^−^ can induce ferroptosis and has been shown to enhance the efficacy of radiotherapy and chemotherapy in multiple tumor models. However, GSCs, especially MES GSCs, can acquire adaptive mechanisms that buffer lipid peroxidation and attenuate ferroptotic cell death. Understanding how MES GSCs suppress ferroptosis may therefore uncover therapeutic vulnerabilities that can be exploited to overcome radioresistance.

Metabolic reprogramming is a hallmark of GBM and plays an active role in shaping tumor cell state, stress tolerance, and therapeutic response. MES GSCs often exhibit enhanced glycolytic activity, leading to elevated lactate production. Traditionally viewed as an end product of aerobic glycolysis, lactate is now recognized as a signaling metabolite that regulates tumor progression, immune evasion, metabolic adaptation, and epigenetic remodeling [14, 15, 16]. One important mechanism by which lactate reshapes gene expression is lysine lactylation, a post‐translational modification of histone and non‐histone proteins [5, 17, 18]. Histone lactylation has been implicated in transcriptional regulation, inflammatory responses, stemness maintenance, and tumor progression [19, 20, 21]. Nevertheless, whether lactate‐driven histone lactylation contributes to GSC radioresistance, and how this modification intersects with ferroptosis regulation, remains poorly understood.

Recent studies have highlighted the importance of membrane lipid remodeling in determining ferroptosis susceptibility. Polyunsaturated phosphatidylethanolamines (PE‐PUFAs) are preferred substrates for lipid peroxidation and are key determinants of ferroptotic cell death, whereas monounsaturated phospholipids are generally less prone to peroxidation [22]. Membrane‐bound O‐acyltransferase domain‐containing 2 (MBOAT2) is a lysophospholipid acyltransferase that preferentially incorporates monounsaturated fatty acids into lyso‐phosphatidylethanolamine, thereby increasing PE‐MUFA abundance and reducing the availability of pro‐ferroptotic PE‐PUFA species. Through this lipid remodeling activity, MBOAT2 can suppress ferroptosis independently of canonical GPX4‐ and FSP1‐dependent antioxidant pathways [22]. However, whether glycolytic reprogramming regulates MBOAT2 expression or activity in MES GSCs, and whether MBOAT2‐mediated lipid remodeling directly contributes to radioresistance, remains unclear.

In this study, we identify a metabolic–epigenetic–lipid remodeling axis that links glycolysis‐derived lactate to ferroptosis suppression and radioresistance in MES GSCs. We show that MES‐associated transcriptional regulators contribute to LDHA upregulation and lactate accumulation in MES GSCs. Lactate promotes p300‐dependent H3K18 lactylation, which enhances USP30 transcription. USP30, in turn, stabilizes MBOAT2 by limiting its ubiquitination, leading to PE‐MUFA remodeling and reduced ferroptosis‐associated lipid peroxidation. Functionally, disruption of this axis enhances ferroptosis, prolongs irradiation‐induced DNA damage persistence, and sensitizes MES GSCs to radiotherapy. Furthermore, pharmacological targeting of glycolysis and USP30 improves radiotherapeutic efficacy in intracranial GSC xenograft models. These findings establish the LDHA–H3K18la–USP30–MBOAT2 axis as a ferroptosis‐protective mechanism in MES GSCs and suggest a potential therapeutic strategy for overcoming GBM radioresistance.

## 2 Materials and Methods

### 2.1 Isolation and Culture of Cells

Glioma stem cells (GSCs) were provided by Dr. Xiuxing Wang from Nanjing Medical University. GSCs were originally isolated from freshly resected human glioblastoma (GBM) specimens or patient‐derived GBM xenografts according to previously established protocols [4, 23]. Briefly, freshly resected GBM tissues were dissociated using the Papain Dissociation System (Worthington Biochemical) according to the manufacturer's instructions. GSCs were cultured as neurospheres in Neurobasal‐A medium supplemented with B27 supplement, 20 ng/mL epidermal growth factor (EGF), 20 ng/mL basic fibroblast growth factor (bFGF), 1 mM L‐glutamine, 100 U/mL penicillin, and 100 µg/mL streptomycin. HEK293T cells were obtained from the Cell Bank of the Chinese Academy of Sciences (Shanghai, China) and maintained in DMEM supplemented with 10% fetal bovine serum.

Molecular classification of GSCs into mesenchymal (MES) or proneural (PN) subtypes was primarily based on RNA‐sequencing‐derived subtype signatures and further validated by subtype‐associated marker expression [24]. WL1, 2907, and MES28 cells were used as MES GSC models, whereas MGG8, PN35, and PN182 cells were used as PN GSC models where indicated. MES GSCs showed higher expression of MES‐associated markers, including CD44, ALDH1A3, C/EBPβ, TAZ, and p‐STAT3, whereas PN GSCs showed relatively higher expression of PN‐associated markers, including OLIG2 and SOX2. The subtype identity of these GSC lines was further confirmed by sphere‐forming capacity and flow cytometric analysis of established MES and PN markers (Figure S1).

All cells were routinely tested for mycoplasma contamination using the MycoAlert PLUS Mycoplasma Detection Kit (Lonza).

### 2.2 Western Blot Analysis

Cells were lysed in RIPA buffer supplemented with protease and phosphatase inhibitors. Protein concentrations were determined using a BCA protein assay kit. Equal amounts of protein were separated by SDS‐PAGE and transferred onto nitrocellulose or PVDF membranes. Membranes were blocked with 5% nonfat milk or 5% BSA in TBST and incubated with the indicated primary antibodies overnight at 4°C. After incubation with HRP‐conjugated secondary antibodies, signals were detected using enhanced chemiluminescence reagents.

For total cellular proteins, GAPDH was used as the loading control. For histone modifications, acid‐extracted histones or histone‐enriched fractions were analyzed, and Histone H3 was used as the loading control. H3K18la and other histone lactylation signals were normalized to total Histone H3. For immunoprecipitated p300 samples, pan‐Kla and pan‐Kac signals were normalized to immunoprecipitated p300. Band intensities were quantified using Image Lab software (Bio‐Rad). Antibody information is provided in Supplementary Table S3.

### 2.3 Extreme Limiting Dilution Assay and Neurosphere Formation

For the in vitro limiting dilution assay, GSCs subjected to the indicated genetic modifications or treatments were dissociated into single cells and seeded into 96‐well plates at 1, 5, 10, 20, or 50 cells per well. Sphere formation was evaluated after 7 days for WL1 and 2907 cells. Wells containing at least one tumor sphere were scored as positive. Stem cell frequency was calculated using the Extreme Limiting Dilution Analysis software (http://bioinf.wehi.edu.au/software/elda).

For primary neurosphere formation, dissociated single cells were plated at the indicated density in GSC medium, and sphere number and diameter were quantified after 7 days. For secondary neurosphere formation, primary spheres were dissociated into single cells and replated under the same conditions. Sphere diameter was measured using ImageJ, and sphere‐forming efficiency was calculated as the number of spheres relative to the number of seeded cells.

### 2.4 Immunohistochemistry and Clinical Tissue Analysis

Human GBM specimens and xenograft tumor tissues were fixed in 4% paraformaldehyde, embedded in paraffin, sectioned, deparaffinized, rehydrated, and subjected to antigen retrieval. Sections were incubated with primary antibodies against LDHA, H3K18la, USP30, MBOAT2, and other indicated markers, followed by HRP‐conjugated secondary antibodies. Signals were visualized using DAB substrate and counterstained with hematoxylin.

For clinical validation, primary and recurrent GBM specimens were analyzed when available. H3K18la staining was evaluated primarily as nuclear staining, whereas LDHA, USP30, and MBOAT2 were evaluated mainly as cytoplasmic staining. IHC intensity and the percentage of positive cells were quantified using H‐score analysis: H‐score = ∑(*I*×*P_i_*), where *I* represents staining intensity and *P_i_* represents the percentage of cells at each intensity level. Quantification was performed by investigators blinded to clinical information.

### 2.5 **γ**‐H2AX Foci Formation Assay

For γ‐H2AX immunofluorescence, GSCs were plated onto poly‐L‐lysine‐ or laminin‐coated chamber slides and allowed to attach overnight. Cells were exposed to 4 Gy ionizing radiation or left untreated and harvested at the indicated time points. For DNA damage repair kinetics, cells were fixed at 0.5, 2, 12, and 24 h after irradiation. Cells were fixed with 4% paraformaldehyde, permeabilized with Triton X‐100, blocked with BSA, and incubated with anti‐γ‐H2AX (Ser139) antibody, followed by Alexa Fluor‐conjugated secondary antibodies. Nuclei were counterstained with DAPI. Images were captured using a fluorescence microscope or confocal microscope. Cells containing more than 10 nuclear γ‐H2AX foci were defined as γ‐H2AX‐positive cells. In selected experiments, γ‐H2AX foci per nucleus were also quantified using ImageJ.

### 2.6 Terminal Deoxynucleotidyl Transferase dUTP Nick End Labeling (TUNEL) Assay

Cells or tissue sections were fixed with 4% paraformaldehyde and subjected to TUNEL staining using the One‐Step TUNEL Apoptosis Assay Kit (Beyotime, C1086) according to the manufacturer's instructions. Images were captured using an Olympus FSX100 microscope. TUNEL‐positive cells were quantified from randomly selected fields.

### 2.7 Flow Cytometry

For apoptosis analysis, cells were collected, washed with PBS, and stained with Annexin V‐FITC and propidium iodide (PI) according to the manufacturer's protocol. For cell cycle or PI‐based analysis, cells were fixed with cold 4% paraformaldehyde, permeabilized with 0.25% Triton X‐100, and stained with PI. Data were acquired using a BD FACS Canto II flow cytometer and analyzed with FlowJo v10 software.

### 2.8 [1,2‐13C]Glucose Metabolic Flux Analysis

MES and PN GSCs were incubated in glucose‐free Neurobasal‐A medium supplemented with B27 and GlutaMAX, followed by labeling with 25 mM [1,2‐^13^C]glucose for 5 h. Cells were washed, collected, and extracted using methanol‐based extraction. Metabolites were dried, reconstituted in ultrapure water, normalized to protein concentration, and analyzed using a high‐resolution mass spectrometry platform. Isotopologue distributions of glycolytic and tricarboxylic acid cycle intermediates were analyzed as indicated.

### 2.9 2‐DG Uptake Assay

GSCs were incubated with Neurobasal‐A/B27/GlutaMAX medium containing 1 mM 2‐deoxyglucose (2‐DG) at 37°C for 20 min. Glucose uptake was assessed using the Glucose Uptake‐Glo Assay Kit (Promega, J1341). Luminescence was measured using a Varioskan LUX plate reader. Background luminescence was determined using lysates from cells not exposed to 2‐DG.

### 2.10 Measurement of Extracellular Acidification Rate (ECAR) and Oxygen Consumption Rate (OCR)

Extracellular acidification rate (ECAR) and oxygen consumption rate (OCR) were measured using a Seahorse XF analyzer. For ECAR analysis, cells were incubated in XF assay medium and sequentially treated with 10 mM glucose, 5 µM oligomycin, and 50 mM 2‐DG. For OCR analysis, cells were sequentially treated with 2 µM oligomycin, 1 µM FCCP, and 1 µM rotenone/antimycin A. Data were normalized to cell number or protein concentration. The ECAR/OCR ratio was calculated using maximal ECAR and maximal OCR values.

### 2.11 Lipid ROS Analysis

Lipid peroxidation was assessed using BODIPY 581/591 C11 (Invitrogen, D3861). GSCs were seeded in six‐well plates and treated as indicated. Cells were then incubated with 1.5 µM BODIPY C11 for 30 min at 37°C, washed with PBS, filtered through a 35‐µm cell strainer, and analyzed by flow cytometry with excitation at 488 nm. Oxidized C11‐BODIPY signals were quantified using FlowJo software.

### 2.12 Lipidomics Analyses

Lipidomic analyses were performed by LipidALL Technologies using an ExionLC‐AD system coupled to a Sciex QTRAP 6500 PLUS mass spectrometer, as previously documented [25, 26]. Lipid classes were separated by normal‐phase high‐performance liquid chromatography on a TUP‐HB silica column. Mobile phase A consisted of chloroform/methanol/ammonium hydroxide, and mobile phase B consisted of chloroform/methanol/ammonium hydroxide/water. Lipid species were quantified using multiple reaction monitoring transitions with spiked internal standards, including DMPC and DMPE.

For lipid rescue experiments, PE‐OA 18:1 and PE‐FA 20:4 were prepared according to the manufacturer's instructions. Cells were pretreated with PE‐OA 18:1 or PE‐FA 20:4 (20 µM, 12 h) before irradiation or ferroptosis‐related treatments. Lipid ROS accumulation was assessed using BODIPY 581/591 C11 staining, and radiation‐induced DNA damage was evaluated by γ‐H2AX immunofluorescence.

### 2.13 Quantitative Real‐Time Polymerase Chain Reaction (qRT‐PCR)

Total RNA was extracted using TRIzol reagent according to the manufacturer's instructions. RNA concentration and purity were determined by spectrophotometry. cDNA was synthesized using the iScript cDNA synthesis kit. qRT‐PCR was performed using SYBR Green‐based detection. GAPDH was used as the internal control. Relative gene expression was calculated using the 2^‐ΔΔCt method. Primer sequences are listed in Supplementary Table S2.

### 2.14 Plasmid Construction, Mutagenesis, and Transfection

Human LDHA, USP30, MBOAT2, ubiquitin, and related sequences were subcloned into pcDNA3.1‐based vectors containing Flag, HA, His, or other indicated tags. Site‐directed mutagenesis of MBOAT2 H373A, USP30 catalytic‐dead mutant C77A, and Histone H3K18R was performed using PCR‐based mutagenesis and verified by Sanger sequencing.

For stable expression of H3WT, H3K18R, MBOAT2‐WT, MBOAT2‐H373A, or other indicated constructs, full‐length sequences were cloned into lentiviral expression vectors and introduced into GSCs, followed by puromycin selection where appropriate. For stable or inducible knockdown, shRNA or miR‐E shRNA sequences targeting p300, LDHA, USP30, MBOAT2, or other indicated genes were cloned into lentiviral vectors, including the LT3GEPIR vector where indicated [27].

For transient knockdown experiments, siRNAs targeting CEBPB, STAT3, or WWTR1/TAZ were transfected using Lipofectamine RNAiMAX or Lipofectamine 3000 according to the manufacturer's instructions. Knockdown efficiency was validated by qRT‐PCR and/or immunoblotting 48–72 h after transfection. At least two independent siRNA or shRNA sequences were used where feasible.

### 2.15 Immunoprecipitation, Co‐Immunoprecipitation, and Ubiquitination Assays

Cells were lysed in IP lysis buffer supplemented with protease inhibitors. Cell lysates were incubated with the indicated antibodies or normal IgG overnight at 4°C, followed by incubation with Protein A/G agarose or magnetic beads. Immunoprecipitates were washed and analyzed by immunoblotting.

For p300 modification assays, endogenous p300 was immunoprecipitated and detected with pan‐Kla, pan‐Kac, and p300 antibodies. Normal IgG was used as a negative control, and pan‐Kla or pan‐Kac signals were normalized to immunoprecipitated p300. For p300–histone H3 interaction assays, p300 was immunoprecipitated and histone H3 was detected by immunoblotting.

For MBOAT2 ubiquitination assays, endogenous or exogenous MBOAT2 was immunoprecipitated and analyzed using anti‐ubiquitin or anti‐HA antibodies. For overexpression assays, HEK293T cells were transfected with Flag‐MBOAT2, His‐USP30 or USP30 mutant, and HA‐ubiquitin constructs. Where indicated, cells were treated with MG132 (20 µM, 8 h) before harvest. Ubiquitination signals were normalized to immunoprecipitated MBOAT2.

### 2.16 In Vitro Lactylation Assay

In vitro lactylation assays were performed using recombinant p300, recombinant histone H3, and lactyl‐CoA. Recombinant histone H3 was incubated with recombinant p300 in lactylation reaction buffer in the presence or absence of lactyl‐CoA. Where indicated, A‐485 was added to inhibit p300/CBP catalytic activity. Reactions were incubated and terminated by adding SDS loading buffer. Reaction products were analyzed by immunoblotting using anti‐H3K18la and anti‐Histone H3 antibodies. H3K18la signals were normalized to total Histone H3. The following reactions were included as controls: H3 alone, H3 plus lactyl‐CoA, H3 plus p300, H3 plus p300 plus lactyl‐CoA, and H3 plus p300 plus lactyl‐CoA plus A‐485.

### 2.17 ChIP‐seq

Cells were cross‐linked with 1% formaldehyde and sonicated to generate chromatin fragments. After pre‐clearing with Protein G agarose, chromatin was incubated overnight with anti‐H3K18la antibody. Protein A/G beads were used to capture antibody–chromatin complexes. DNA was eluted, reverse‐cross‐linked, purified, and used for library preparation. ChIP‐seq libraries were sequenced on the HiSeq platform by LC‐Bio. Sequencing reads were aligned to the human reference genome, and peaks were identified using standard peak‐calling pipelines.

### 2.18 Chromatin Immunoprecipitation‐Quantitative PCR (ChIP‐qPCR)

ChIP‐qPCR was performed using the BeyoChIP ChIP Assay Kit according to the manufacturer's instructions. Briefly, cells were cross‐linked with formaldehyde, lysed, and sonicated to generate chromatin fragments. Chromatin was immunoprecipitated using antibodies against H3K18la, p300, C/EBPβ, STAT3, p‐STAT3, TAZ, TEAD4, or normal IgG.

For LDHA promoter analysis, candidate binding regions for C/EBPβ, STAT3, and TEAD/TAZ‐associated regulatory elements were predicted using JASPAR motif analysis. ChIP‐qPCR primers were designed to amplify regions flanking the predicted motifs, including P1 for the C/EBPβ‐associated site, P2 for the STAT3‐associated site, P3 for the TEAD/TAZ‐associated site, and P4 as a negative control region.

For USP30 promoter analysis, H3K18la and p300 enrichment at the USP30 promoter was quantified by qPCR using primers spanning the predicted H3K18la‐enriched promoter region. ChIP‐qPCR data were presented as percentage of input or fold enrichment over IgG.

### 2.19 USP30 Promoter Luciferase Reporter Assay

The human USP30 promoter region was amplified and cloned upstream of the firefly luciferase reporter gene. Cells were co‐transfected with the USP30 promoter luciferase construct and a Renilla luciferase plasmid as an internal control. After transfection, cells were treated with indicated interventions. Luciferase activity was measured using a dual‐luciferase reporter assay system. Firefly luciferase activity was normalized to Renilla luciferase activity. Relative luciferase activity was expressed as fold change over the control group.

### 2.20 Clonogenic Survival Assay

GSCs were dissociated into single cells, treated with the indicated compounds or genetic modifications, and exposed to increasing doses of ionizing radiation. Cells were then plated at appropriate densities in GSC medium and allowed to form colonies or spheres. After incubation, colonies/spheres were counted, and surviving fractions were calculated relative to the non‐irradiated control. Survival curves were fitted using the linear–quadratic model.

### 2.21 TCGA, CGGA, and Clinical Cohort Analyses

Transcriptomic and clinical data from TCGA‐GBM and CGGA‐GBM cohorts were analyzed to assess the clinical relevance of LDHA, USP30, and MBOAT2. Gene expression values were log‐transformed where appropriate. The MES signature score was calculated by averaging the expression values of Verhaak MES signature genes. Pairwise correlations among LDHA, USP30, MBOAT2, and MES signature scores were evaluated using Spearman correlation analysis.

For survival analysis, a composite transcriptomic axis score was calculated based on the expression of LDHA, USP30, and MBOAT2. Patients were stratified into high‐ and low‐axis groups using the median value or an optimal cutpoint, as indicated. For radiotherapy‐treated TCGA and CGGA survival analyses, the optimal cutpoint of the LDHA–USP30–MBOAT2 axis score was used. Kaplan–Meier curves were generated, and statistical significance was assessed using the log‐rank test.

Because H3K18la is a histone post‐translational modification and cannot be directly evaluated from transcriptomic datasets, protein‐level validation was performed using an in‐house GBM cohort. IHC staining of LDHA, H3K18la, USP30, and MBOAT2 was quantified using H‐scores. Associations between protein expression, recurrence status, and patient survival were analyzed using Mann–Whitney U test, Spearman correlation analysis, Kaplan–Meier analysis, and log‐rank test, as appropriate.

### 2.22 Xenograft Mouse Model

Six‐week‐old male nude mice were obtained from the Animal Center of Nanjing Medical University. For intracranial xenograft experiments, GSCs were dissociated into single cells, and 1 × 10^4^ cells suspended in PBS were stereotactically implanted into the right cerebral cortex at a depth of 3.5 mm. Tumor growth was monitored using the IVIS Imaging System, and bioluminescence signals were quantified using Living Image software.

For therapeutic studies, tumor‐bearing mice were randomized into the indicated treatment groups after tumor establishment. Oxamate was administered intraperitoneally, and USP30‐IN‐20 was administered by oral gavage. For the optimized therapeutic regimen, oxamate was given at 300 mg/kg once daily, and USP30‐IN‐20 was given at 10 mg/kg once daily. Local cranial irradiation was delivered as 2.5 Gy per fraction for four consecutive days. For dose‐optimization experiments, low‐, intermediate‐, and high‐dose regimens corresponded to oxamate at 150, 300, and 450 mg/kg, respectively, combined with USP30‐IN‐20 at 5, 10, and 15 mg/kg, respectively. For treatment‐schedule optimization, synchronous administration and oxamate‐pretreated sequential administration were compared as indicated.

Body weight and neurological status were monitored during treatment and follow‐up. For toxicity evaluation, non‐tumor brain tissues were collected for H&E staining where indicated. Tumor tissues were collected for histological analysis, immunohistochemistry, and transmission electron microscopy according to the experimental design.

All animal experiments were approved by the Animal Experimental Ethics Committee of Nanjing Medical University and were conducted in accordance with institutional and national guidelines.

### 2.23 Statistical Analyses

Statistical analyses were performed using GraphPad Prism 8.0.2. Data are presented as mean ± SD or mean ± SEM, as indicated in figure legends. Comparisons between two groups were performed using unpaired two‐tailed Student's t‐test or Mann–Whitney U test, depending on data distribution. Comparisons among multiple groups were performed using one‐way ANOVA followed by Tukey's post hoc test. Time‐course experiments, including γ‐H2AX foci kinetics and tumor growth curves, were analyzed using two‐way ANOVA with appropriate multiple‐comparison correction. Kaplan–Meier survival curves were compared using the log‐rank test. Spearman correlation analysis was used to evaluate associations between gene or protein expression levels. P < 0.05 was considered statistically significant.

## 3 Results

### 3.1 Ferroptosis Activation Restrains the MES Phenotype and Self‐Renewal Capacity of GSCs

**FIGURE 1:**
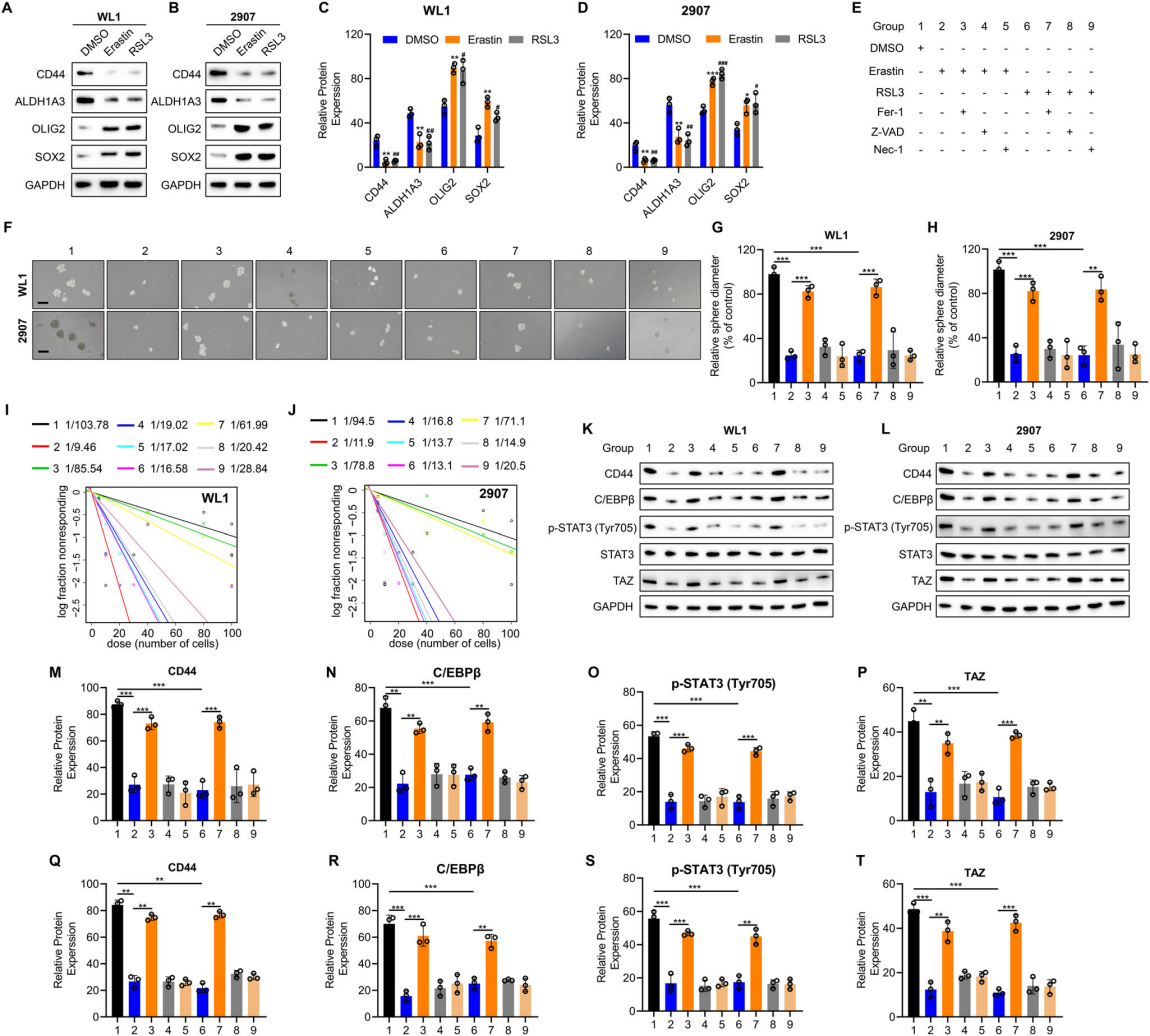
Ferroptosis activation restrains the MES phenotype and self‐renewal capacity of GSCs. (A,B) Immunoblot analysis of CD44, ALDH1A3, OLIG2, and SOX2 in WL1 (A) and 2907 (B) MES GSCs treated with DMSO, Erastin, or RSL3. GAPDH was used as the loading control. Cells were treated with Erastin (5 µM, 6 h) or RSL3 (0.1 µM, 24 h). (C,D) Densitometric quantification of the Western blots shown in (A, B). CD44, ALDH1A3, OLIG2, and SOX2 protein levels were normalized to GAPDH. Data are shown as mean ± SEM from *n* = 3 independent biological experiments. *p < 0.05, **p < 0.01, ***p < 0.001 versus DMSO for Erastin‐treated cells; #*p* < 0.05, ##*p* < 0.01, ###*p* < 0.001 versus DMSO for RSL3‐treated cells. (E) Treatment scheme for the sphere formation, limiting dilution assays and immunoblot analysis. Groups were as follows: 1, DMSO; 2, Erastin; 3, Erastin + Fer‐1; 4, Erastin + Z‐VAD‐FMK; 5, Erastin + Nec‐1; 6, RSL3; 7, RSL3 + Fer‐1; 8, RSL3 + Z‐VAD‐FMK; 9, RSL3 + Nec‐1. Cells were treated with Erastin (5 µM, 6 h), RSL3 (0.1 µM, 24 h), Fer‐1 (1 µM, 24 h), Z‐VAD‐FMK (10 µM, 24 h), or Nec‐1 (1 µM, 24 h) as indicated. (F) Representative tumorsphere images of WL1 and 2907 GSCs under the indicated treatment conditions. Scale bar, 25 µm. (G,H) Quantification of relative sphere diameter in WL1 (G) and 2907 (H) GSCs. Sphere diameter was normalized to the DMSO control group. Data are shown as mean ± SEM from *n* = 3 independent biological experiments. Statistical significance was determined by one‐way ANOVA followed by Tukey's multiple‐comparison test. (I,J) Extreme limiting dilution assay of WL1 (I) and 2907 (J) GSCs under the indicated treatment conditions. Stem cell frequency is presented as 1/x with 95% confidence intervals. Statistical significance was calculated using ELDA software. (K,L) Immunoblot analysis of CD44, C/EBPβ, p‐STAT3 (Tyr705), total STAT3, and TAZ in tumorspheres derived from WL1 (K) and 2907 (L) GSCs under the treatment conditions defined in panel E. GAPDH was used as the loading control. (M–T) Densitometric quantification of the Western blot data shown in (K,L). Panels M–P show quantification of CD44 (M), C/EBPβ (N), p‐STAT3 (O), and TAZ (P) in WL1 tumorspheres. Panels Q–T show quantification of CD44 (Q), C/EBPβ (R), p‐STAT3 (S), and TAZ (T) in 2907 tumorspheres. CD44, C/EBPβ, and TAZ were normalized to GAPDH, whereas p‐STAT3 was normalized to total STAT3. Data are presented as mean ± SEM from *n* = 3 independent biological experiments. Statistical significance was determined by one‐way ANOVA followed by Tukey's multiple‐comparison test. *p < 0.05, **p < 0.01, ***p < 0.001; ns, not significant.

To determine whether ferroptosis regulates MES‐associated phenotypes in GSCs, we first examined the expression of MES‐associated markers CD44 and ALDH1A3, as well as PN‐associated markers OLIG2 and SOX2, in two MES GSC lines, WL1 and 2907. Treatment with the ferroptosis inducers Erastin or RSL3 markedly decreased CD44 and ALDH1A3 expression, whereas OLIG2 and SOX2 expression was increased (Figure 1A–D). These results suggest that ferroptosis activation attenuates the MES‐associated marker profile in MES GSCs.

We next assessed whether ferroptosis activation affects the self‐renewal capacity of MES GSCs. Erastin or RSL3 treatment reduced tumorsphere size and sphere‐forming frequency in both WL1 and 2907 cells (Figure 1E–J). Importantly, the reduction in sphere formation was largely rescued by the ferroptosis inhibitor Ferrostatin‐1 (Fer‐1), but not by the apoptosis inhibitor Z‐VAD‐FMK or the necroptosis inhibitor Necrostatin‐1 (Nec‐1) (Figure 1F–J). These findings indicate that impaired sphere‐forming capacity is primarily associated with ferroptosis activation rather than apoptosis or necroptosis.

To further validate the effect of ferroptosis on MES‐state maintenance, we analyzed MES‐associated transcriptional regulators in tumorspheres collected from the indicated treatment groups. Immunoblotting showed that Erastin or RSL3 decreased the protein levels of CD44, C/EBPβ, p‐STAT3, and TAZ, whereas Fer‐1 restored the expression of these MES‐associated markers. In contrast, Z‐VAD‐FMK or Nec‐1 did not produce a comparable rescue effect (Figure 1K–T). Together, these results demonstrate that ferroptosis activation suppresses MES‐associated phenotypes and self‐renewal capacity in MES GSCs.

### 3.2 Ferroptosis Activation Impairs the Radioresistance of MES GSCs

**FIGURE 2:**
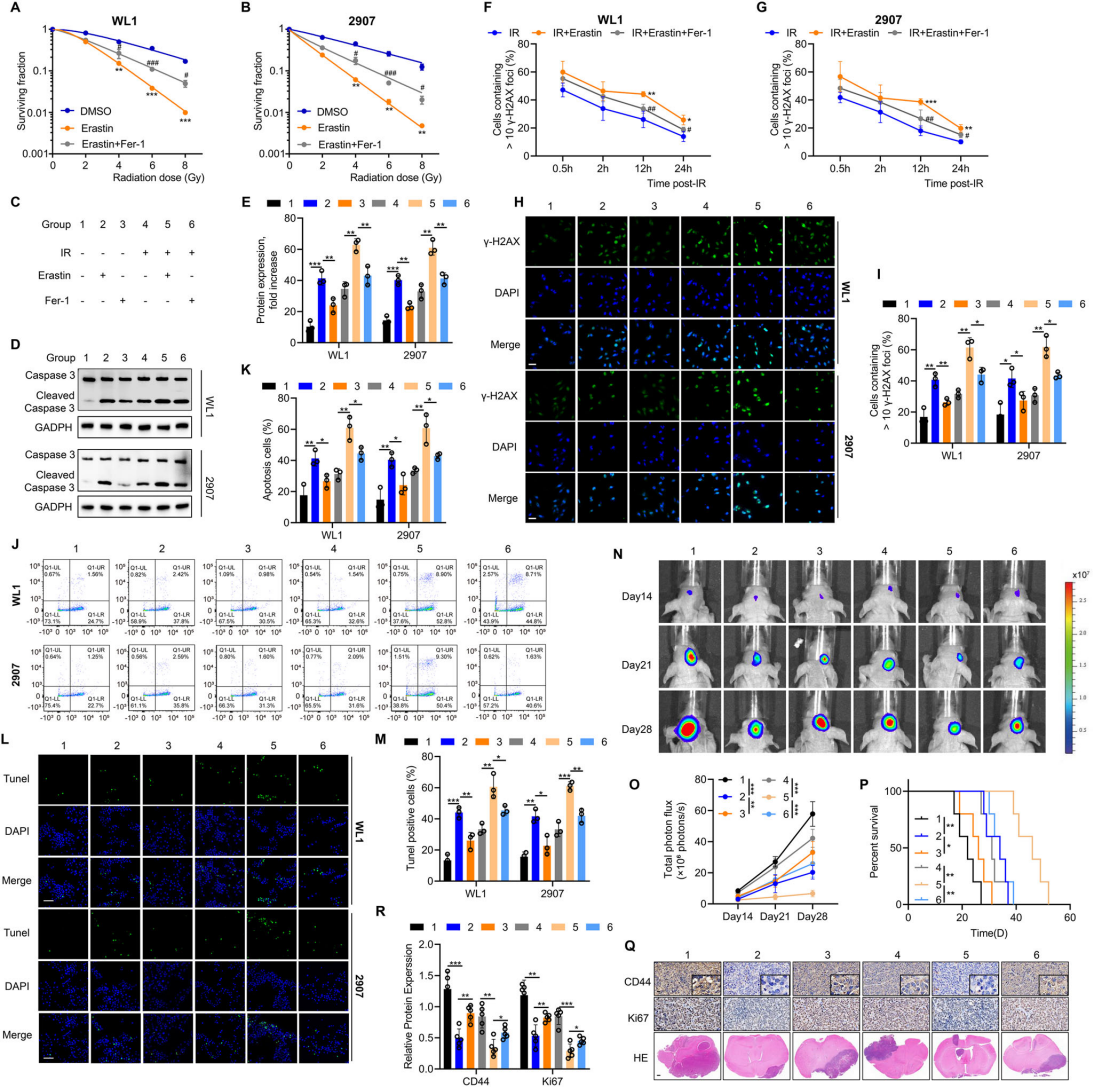
Ferroptosis activation impairs the radioresistance of MES GSCs. (A,B) Clonogenic survival curves of WL1 (A) and 2907 (B) MES GSCs after exposure to increasing doses of ionizing radiation. Cells were treated with DMSO, Erastin, or Erastin plus Fer‐1 before irradiation. Erastin (5 µM, 6 h) enhanced radiosensitivity, whereas Fer‐1 (1 µM, 24 h) partially rescued clonogenic survival. Data are presented as mean ± SEM from *n* = 3 independent biological experiments. Survival curves were fitted using the linear–quadratic model. *p < 0.05, **p < 0.01, ***p < 0.001 versus DMSO; #*p* < 0.05, ##*p* < 0.01, ###*p* < 0.001 versus Erastin. (C) Treatment scheme for in vitro irradiation experiments. Groups were as follows: 1, DMSO; 2, IR; 3, IR + Erastin; 4, IR + Erastin + Fer‐1; 5, Erastin; 6, Erastin + Fer‐1. Cells were irradiated with 4 Gy X‐rays at a dose rate of 2 Gy/min where indicated. Erastin was used at 5 µM for 6 h, and Fer‐1 was used at 1 µM for 24 h. (D) Immunoblot analysis of total caspase‐3 and cleaved caspase‐3 in WL1 and 2907 GSCs under the treatment conditions defined in (C). GAPDH was used as the loading control. (E) Densitometric quantification of cleaved caspase‐3 protein levels shown in (D). Cleaved caspase‐3 was normalized to GAPDH. Data are shown as mean ± SEM from *n* = 3 independent biological experiments. Statistical significance was determined by one‐way ANOVA followed by Tukey's multiple‐comparison test. (F,G) Time‐course quantification of γ‐H2AX‐positive cells in WL1 (F) and 2907 (G) GSCs treated with IR, IR + Erastin, or IR + Erastin + Fer‐1. Cells were fixed at 0.5, 2, 12, and 24 h after 4 Gy irradiation. γ‐H2AX‐positive cells were defined as cells containing more than 10 nuclear γ‐H2AX foci. Data are presented as mean ± SEM from *n* = 3 independent biological experiments. Statistical significance was determined by two‐way ANOVA followed by Tukey's multiple‐comparison test. *p < 0.05, **p < 0.01, ***p < 0.001 versus IR; #*p* < 0.05, ##*p* < 0.01, ###*p* < 0.001 versus IR + Erastin. (H) Representative γ‐H2AX immunofluorescence images of WL1 and 2907 GSCs at 12 h after 4 Gy irradiation under the treatment conditions defined in (C). Nuclei were counterstained with DAPI. Scale bar, 50 µm. (I) Quantification of γ‐H2AX‐positive cells from the representative 12 h γ‐H2AX immunofluorescence experiment shown in (H). Data are shown as mean ± SEM from *n* = 3 independent biological experiments. Statistical significance was determined by one‐way ANOVA followed by Tukey's multiple‐comparison test. (J) Representative Annexin V/PI flow cytometry plots of WL1 and 2907 GSCs under the treatment conditions defined in (C). (K) Quantification of apoptotic cells from the flow cytometry analysis shown in (J). Apoptotic cells were defined as Annexin V‐positive cells. Data are shown as mean ± SEM from *n* = 3 independent biological experiments. (L) Representative TUNEL staining images of WL1 and 2907 GSCs under the treatment conditions defined in (C). Nuclei were counterstained with DAPI. Scale bar, 100 µm. (M) Quantification of TUNEL‐positive cells shown in (L). Data are shown as mean ± SEM from *n* = 3 independent biological experiments. (N) Representative bioluminescence images of intracranial WL1 GSC xenografts at the indicated time points after treatment. Mice were treated with vehicle, Erastin, Fer‐1, irradiation, or the indicated combinations. Erastin was administered intraperitoneally at 10 mg/kg every other day for 2 weeks. Fer‐1 was administered intraperitoneally at 5 mg/kg 1 h before Erastin in the rescue groups. Local cranial irradiation was delivered at 2.5 Gy per fraction once daily for four consecutive days, for a total dose of 10 Gy, at a dose rate of 2 Gy/min. *n* = 5 male nude mice per group. (O) Quantification of intracranial tumor burden based on IVIS bioluminescence imaging. Total photon flux within a fixed brain ROI was measured on days 14, 21, and 28 after WL1 GSC implantation. Groups 1–6 correspond to the treatment conditions defined in panel C. Data are shown as mean ± SEM, n = 5 male nude mice per group. Statistical significance was determined by two‐way ANOVA with Tukey's post hoc test. (P) Kaplan–Meier survival analysis of mice bearing intracranial WL1 GSC xenografts under the indicated treatment conditions. n = 5 male nude mice per group. Statistical significance was determined by log‐rank test. (Q) Representative IHC staining for CD44 and Ki67 and H&E staining of intracranial WL1 GSC‐derived xenograft tumors under the indicated treatment conditions. Scale bars, 25 µm for IHC and 1 mm for H&E. (R) Quantification of CD44 and Ki67 staining in xenograft tumors shown in (Q). Data are presented as mean ± SEM from *n* = 5 male nude mice per group. For all bar graphs, data are presented as mean ± SEM unless otherwise stated. *p < 0.05, **p < 0.01, ***p < 0.001; ns, not significant.

Maintenance of the MES state has been closely linked to radioresistance in GSCs [4, 28]. Given that ferroptosis activation restrained MES‐associated phenotypes, we next examined whether ferroptosis affects the radiosensitivity of MES GSCs. Because Erastin and RSL3 produced comparable effects on MES marker expression, Erastin was selected for subsequent radiosensitivity assays. Clonogenic survival analysis showed that Erastin markedly reduced the surviving fraction of irradiated WL1 and 2907 MES GSCs, whereas the ferroptosis inhibitor Ferrostatin‐1 (Fer‐1) partially restored clonogenic survival (Figure 2A,B). These results suggest that ferroptosis activation sensitizes MES GSCs to irradiation. WL1 and 2907 cells. We next assessed whether ferroptosis activation affects radiation‐induced DNA damage kinetics. Time‐course analysis of γ‐H2AX foci was performed at 0.5, 2, 12, and 24 h after irradiation. At early time points, especially 0.5 h and 2 h, the proportion of γ‐H2AX‐positive cells was comparable among treatment groups. However, Erastin significantly prolonged γ‐H2AX foci persistence at 12 and 24 h after irradiation, and this effect was partially reversed by Fer‐1 (Figure 2F–I). These findings indicate that ferroptosis activation delays the resolution of radiation‐induced DNA damage rather than simply increasing initial DNA damage formation. Consistent with this enhanced radiosensitivity, Erastin increased cleaved caspase‐3 levels, Annexin V‐positive apoptotic cells, and TUNEL‐positive cells after irradiation, whereas Fer‐1 attenuated these effects (Figure 2C–E,J–M). Thus, ferroptosis activation enhances irradiation‐induced DNA damage persistence and cell death in MES GSCs.

We further evaluated the impact of ferroptosis activation on radiotherapy response in an intracranial WL1 GSC xenograft model. Mice bearing intracranial WL1 GSC xenografts were treated with Erastin to pharmacologically activate ferroptosis, with or without Fer‐1 rescue, followed by fractionated cranial irradiation. Combined Erastin and irradiation markedly suppressed intracranial tumor growth, reduced CD44 and Ki67 staining in tumor tissues, and prolonged mouse survival compared with irradiation alone. Fer‐1 partially reversed the antitumor effect of Erastin combined with irradiation (Figure 2N–Q). Together, these data demonstrate that ferroptosis activation attenuates radioresistance in MES GSCs both in vitro and in vivo.

### 3.3 MES GSCs Exhibit Enhanced Glycolysis and Lactate Production

**FIGURE 3:**
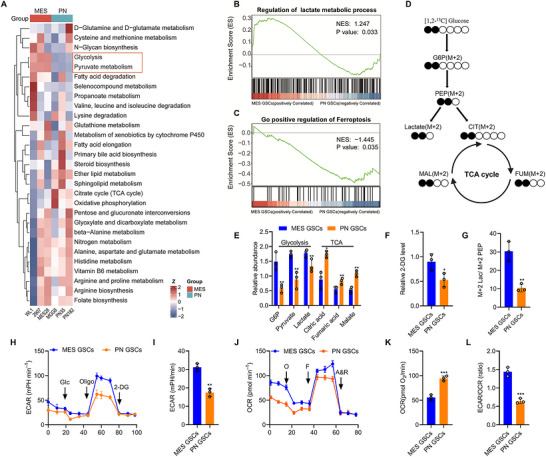
MES GSCs exhibit enhanced glycolysis and lactate production. (A) Targeted metabolomics‐based pathway analysis comparing MES and PN GSCs. Heatmap shows differentially enriched metabolic pathways between MES GSCs and PN GSCs. Data were normalized and presented as z‐scores. (B,C) Gene set enrichment analysis showing enrichment of lactate metabolic process (B) and negative regulation of ferroptosis (C) in MES versus PN GSCs. NES and nominal *p* values are shown. (D) Schematic illustration of [1,2‐^1^ ^3^C]glucose tracing into glycolytic intermediates and TCA cycle metabolites. ^1^ ^3^C‐labeled carbons are indicated as filled circles. (E) Relative abundance of glycolytic metabolites and TCA cycle intermediates derived from [1,2‐^1^ ^3^C]glucose tracing in MES and PN GSCs. Glycolytic metabolites included G6P, pyruvate, and lactate, whereas TCA cycle intermediates included citrate, fumarate, and malate. (F) Glucose uptake in MES and PN GSCs measured using a 2‐DG uptake assay. (G) Ratio of M+2 lactate to M+2 phosphoenolpyruvate derived from [1,2‐^1^ ^3^C]glucose tracing in MES and PN GSCs. (H, I) ECAR trace (H) and quantification of maximal ECAR (I) in MES and PN GSCs following sequential injection of glucose, oligomycin, and 2‐DG. (J,K) OCR trace (J) and quantification of basal OCR (K) in MES and PN GSCs following sequential injection of oligomycin, FCCP, and rotenone/antimycin A. (L) ECAR/OCR ratio in MES and PN GSCs under basal metabolic conditions. Data are presented as mean ± SEM from *n* = 3 independent biological experiments, unless otherwise indicated. Statistical significance was determined by unpaired two‐tailed Student's t‐test. *p < 0.05, **p < 0.01, ***p < 0.001.

Recent studies have shown that metabolic pathways, including glycolysis, the pentose phosphate pathway, and the tricarboxylic acid cycle, can regulate ferroptosis sensitivity [8, 29, 30]. To define the metabolic features associated with the MES state in GSCs, we performed targeted metabolomic profiling of MES and PN GSCs. Pathway analysis revealed that MES GSCs were characterized by enhanced glycolysis and lactate‐associated metabolism compared with PN GSCs (Figure 3A). Consistently, GSEA showed that lactate metabolic processes were positively enriched in MES GSCs, whereas genes associated with negative regulation of ferroptosis were also enriched in MES GSCs (Figure 3B,C), suggesting a potential link between lactate metabolism and ferroptosis resistance.

To further trace glucose utilization, MES and PN GSCs were cultured with [1,2‐^1^ ^3^C]glucose and analyzed by mass spectrometry (Figure 3D). MES GSCs showed increased abundance of glycolytic metabolites, including G6P, pyruvate, and lactate, whereas PN GSCs displayed relatively higher levels of TCA cycle intermediates, including citrate, fumarate, and malate (Figure 3E). In agreement with these findings, 2‐DG uptake assays demonstrated greater glucose uptake in MES GSCs than in PN GSCs (Figure 3F). Moreover, the ratio of M+2 lactate to M+2 phosphoenolpyruvate was increased in MES GSCs, indicating enhanced conversion of glycolytic carbon into lactate (Figure 3G). We next examined cellular bioenergetics using Seahorse analysis. MES GSCs displayed higher extracellular acidification rates and lower oxygen consumption rates than PN GSCs (Figure 3H–K). Accordingly, the ECAR/OCR ratio was significantly elevated in MES GSCs, indicating a stronger reliance on aerobic glycolysis relative to oxidative phosphorylation (Figure 3L). Together, these metabolic flux and bioenergetic analyses demonstrate that MES GSCs preferentially engage glycolysis and lactate production compared with PN GSCs.

To further determine whether lactate directly regulates ferroptosis sensitivity, PN GSCs were supplemented with exogenous lactate to mimic the lactate‐rich metabolic state observed in MES GSCs. Lactate supplementation partially rescued the reduction in cell viability induced by Erastin or RSL3 in MGG8 and PN35 cells and reduced C11‐BODIPY lipid ROS accumulation (Figure S2A–E). Conversely, pharmacological inhibition of LDHA with stiripentol enhanced Erastin‐ or RSL3‐induced loss of cell viability and lipid ROS accumulation in WL1 and 2907 MES GSCs (Figure S2F–J). These results suggest that lactate negatively regulates ferroptosis sensitivity in GSCs, whereas LDHA inhibition promotes ferroptosis‐associated lipid peroxidation in MES GSCs.

### 3.4 LDHA Inhibition Sensitizes MES GSCs to Irradiation Through Ferroptosis

**FIGURE 4:**
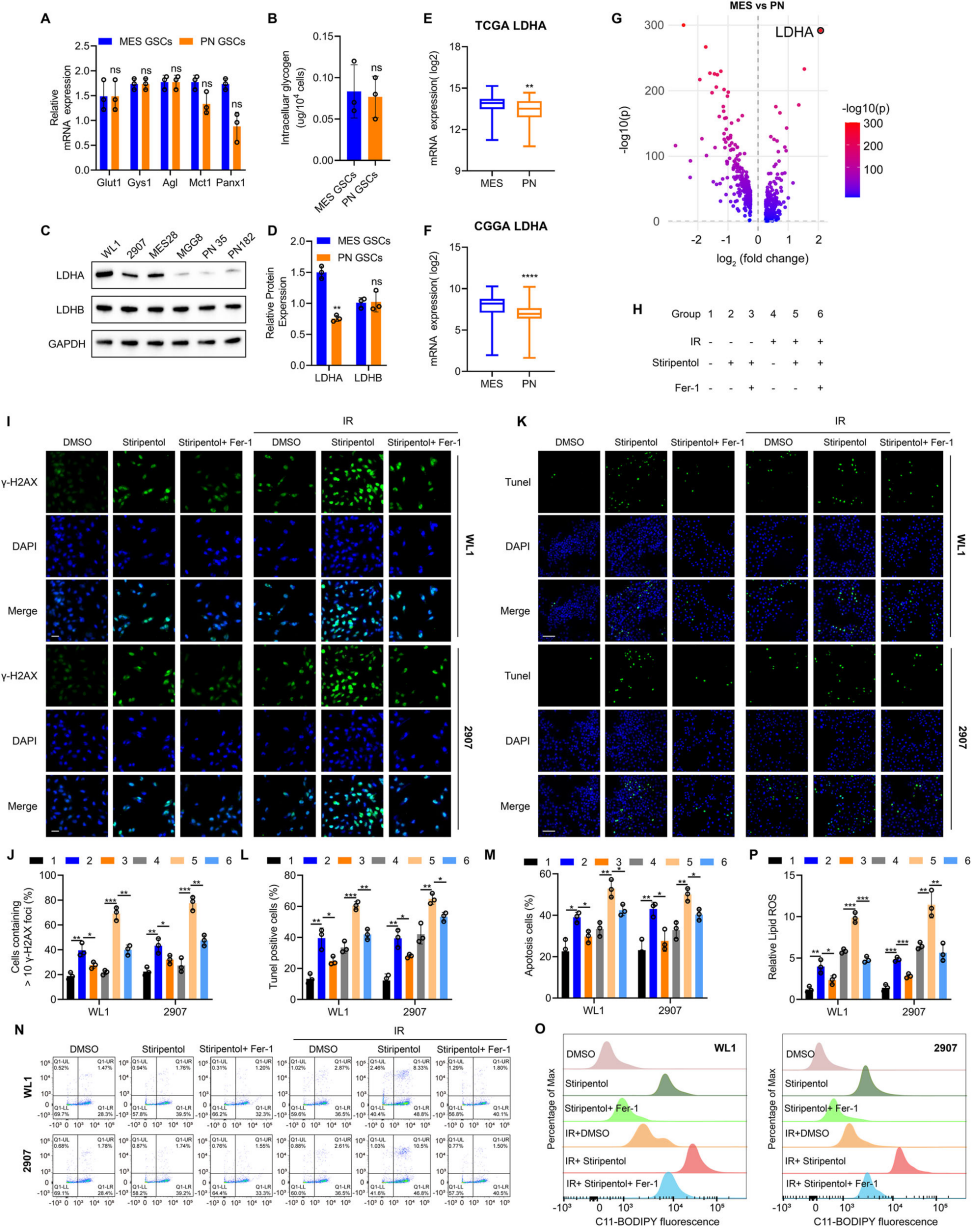
LDHA inhibition sensitizes MES GSCs to irradiation through ferroptosis. (A) qRT‐PCR analysis of genes involved in glucose uptake, glycogen metabolism, and lactate transport in MES and PN GSCs, including GLUT1, GYS1, AGL, MCT1, and PANX1. (B) Intracellular glycogen content in MES and PN GSCs. (C,D) Immunoblot analysis (C) and quantification (D) of LDHA and LDHB protein expression in MES and PN GSCs. GAPDH was used as the loading control. (E,F) LDHA mRNA expression in MES‐ and PN‐subtype GBM samples from TCGA (E) and CGGA (F) datasets. (G) Volcano plot showing differentially expressed genes between MES and PN GSCs. LDHA is highlighted. (H) Treatment scheme for in vitro irradiation experiments. Groups were as follows: 1, DMSO; 2, stiripentol; 3, stiripentol + Fer‐1; 4, IR + DMSO; 5, IR + stiripentol; 6, IR + stiripentol + Fer‐1. (I,J) Representative γ‐H2AX immunofluorescence images (I) and quantification of γ‐H2AX‐positive cells (J) in WL1 and 2907 cells under the indicated treatments. Cells containing more than 10 nuclear γ‐H2AX foci were defined as γ‐H2AX‐positive cells. (K,L) Representative TUNEL staining images (K) and quantification of TUNEL‐positive cells (L) in WL1 and 2907 cells under the indicated treatments. (M,N) Quantification (M) and representative flow cytometry plots (N) of Annexin V/PI apoptosis analysis in WL1 and 2907 cells under the indicated treatments. (O,P) Representative C11‐BODIPY flow cytometry histograms (O) and quantification of relative lipid ROS levels (P) in WL1 and 2907 cells under the indicated treatments. Stiripentol was used at 0.5 mM for 24 h, and Fer‐1 was used at 1 µM for 24 h. Cells were irradiated with 4 Gy at a dose rate of 2 Gy/min where indicated. γ‐H2AX foci and lipid ROS were analyzed at 12 h post‐irradiation, and TUNEL and apoptosis assays were performed at 24 h post‐irradiation. BODIPY 581/591 C11 was used at 1.5 µM for 30 min. Scale bars, 50 µm for γ‐H2AX and 100 µm for TUNEL. Data are presented as mean ± SEM from *n* = 3 independent biological experiments. Statistical significance was determined by unpaired two‐tailed Student's t‐test or one‐way ANOVA followed by Tukey's multiple‐comparison test, as appropriate. *p < 0.05, **p < 0.01, ***p < 0.001; ns, not significant.

We next investigated the mechanism responsible for increased lactate production in MES GSCs. MES and PN GSCs showed no significant differences in intracellular glycogen content or in the expression of selected genes involved in glucose uptake, glycogen metabolism, and lactate transport, including GLUT1, GYS1, AGL, MCT1, and PANX1 (Figure 4A,B). Transcriptomic analysis comparing MES and PN GSCs identified LDHA, which catalyzes the conversion of pyruvate to lactate, as one of the most prominently upregulated metabolic genes in MES GSCs (Figure 4G). Immunoblotting further confirmed higher LDHA protein expression in MES GSCs, whereas LDHB showed no comparable increase (Figure 4C,D). Consistently, TCGA and CGGA analyses showed higher LDHA expression in MES‐subtype GBM tumors than in PN‐subtype tumors (Figure 4E,F). These results suggest that LDHA upregulation is a major contributor to enhanced lactate production in MES GSCs.

To further determine why LDHA is preferentially upregulated in MES GSCs, we examined MES‐associated transcriptional regulators. Knockdown of C/EBPβ, STAT3, or TAZ reduced LDHA mRNA and protein expression and decreased intracellular lactate levels in WL1 and 2907 cells (Figure S3A–M). Promoter motif analysis identified putative C/EBPβ, STAT3, and TEAD/TAZ‐associated binding regions within the LDHA promoter. ChIP‐qPCR further confirmed enrichment of C/EBPβ, p‐STAT3, and TEAD/TAZ at the predicted LDHA promoter regions, whereas the optional control region showed minimal enrichment (Figure S3N–P). These findings indicate that MES‐associated transcription factors directly contribute to LDHA transcriptional activation in MES GSCs.

We then tested whether LDHA inhibition affects the radiosensitivity of MES GSCs. WL1 and 2907 cells were treated with the LDHA inhibitor stiripentol, followed by irradiation where indicated (Figure 4H). γ‐H2AX immunofluorescence showed that stiripentol increased the persistence of radiation‐induced γ‐H2AX foci, and this effect was partially reversed by Fer‐1 (Figure 4I,J). Consistently, stiripentol increased TUNEL‐positive cells and Annexin V‐positive apoptotic cells after irradiation, whereas Fer‐1 attenuated these effects (Figure 4K–N). LDHA inhibition also markedly increased C11‐BODIPY lipid ROS accumulation, which was reduced by Fer‐1 treatment (Figure 4O,P). Together, these data indicate that LDHA inhibition sensitizes MES GSCs to irradiation, at least in part, by enhancing ferroptosis‐associated lipid peroxidation and prolonging radiation‐induced DNA damage persistence.

### 3.5 MBOAT2 Dictates Ferroptosis Sensitivity by Remodeling Cellular Lipid Composition in GSCs

**FIGURE 5:**
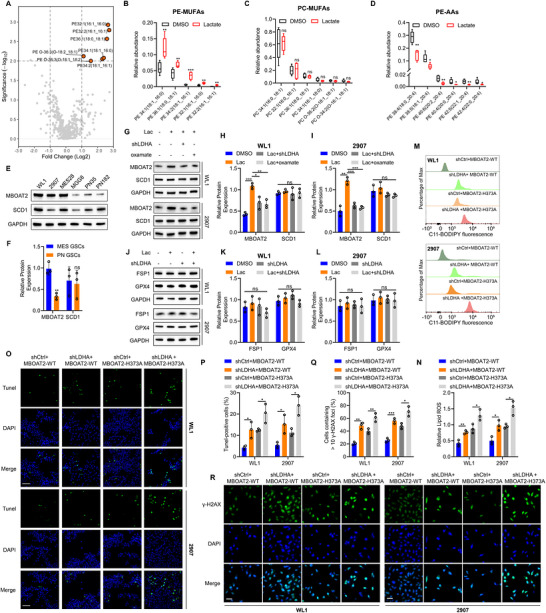
MBOAT2 dictates ferroptosis sensitivity by remodeling cellular lipid composition in GSCs. (A) Volcano plot showing lipid species altered by lactate treatment in WL1 MES GSCs. Cells were treated with lactate (12 mM, 24 h) before lipidomic analysis. Lipid species with fold change >2 and p < 0.01 were considered significantly altered. *n* = 5 biological replicates. (B–D) Relative abundance of representative PE‐MUFAs (B), PC‐MUFAs (C), and PE‐AA species (D) in WL1 cells treated with DMSO or lactate (12 mM, 24 h). n = 5 biological replicates. (E,F) Immunoblot analysis (E) and densitometric quantification (F) of MBOAT2 and SCD1 protein expression in MES and PN GSCs. GAPDH was used as the loading control. (G–I) Immunoblot analysis (G) and quantification (H, I) of MBOAT2 and SCD1 in WL1 and 2907 MES GSCs treated with DMSO, lactate, lactate plus shLDHA, or lactate plus oxamate. Lactate was used at 12 mM for 24 h, and oxamate was used at 20 mM for 24 h. GAPDH was used as the loading control. (J–L) Immunoblot analysis (J) and quantification (K,L) of FSP1 and GPX4 in WL1 and 2907 MES GSCs under the indicated treatments. GAPDH was used as the loading control. (M,N) Representative C11‐BODIPY flow cytometry histograms (M) and lipid ROS quantification (N) in WL1 and 2907 MES GSCs expressing wild‐type MBOAT2 or the catalytically impaired MBOAT2‐H373A mutant under control or LDHA‐depleted conditions after irradiation. Cells were exposed to 4 Gy irradiation and analyzed at 12 h post‐IR. Lipid ROS was detected using BODIPY 581/591 C11 (1.5 µM, 30 min). (O,P) Representative TUNEL staining images (O) and quantification of TUNEL‐positive cells (P) in the indicated cells after 4 Gy irradiation. Cells were analyzed at 24 h post‐IR. Scale bar, 100 µm. (Q,R) Representative γ‐H2AX immunofluorescence images (Q) and quantification of γ‐H2AX‐positive cells (R) in the indicated cells after 4 Gy irradiation. Cells were fixed at 12 h post‐IR. γ‐H2AX‐positive cells were defined as cells containing more than 10 nuclear γ‐H2AX foci. Scale bar, 50 µm. Data are shown as mean ± SEM from n = 3 independent biological experiments, unless otherwise indicated. Statistical significance was determined by two‐tailed Student's t‐test or one‐way ANOVA followed by Tukey's multiple‐comparison test, as appropriate. *p < 0.05, **p < 0.01, ***p < 0.001; ns, not significant.

Polyunsaturated fatty acid‐containing phospholipids are highly susceptible to lipid peroxidation and are critical determinants of ferroptosis sensitivity, whereas monounsaturated fatty acid‐containing phospholipids are relatively resistant to peroxidation [31, 32, 33]. To determine whether lactate alters membrane lipid composition in MES GSCs, we performed lipidomic profiling in WL1 cells treated with or without exogenous lactate. Lactate treatment markedly reshaped the cellular lipidome. Among the altered lipid species, PE‐MUFAs were prominently increased, whereas several PE‐PUFA species were reduced after lactate treatment (Figure 5A). We then examined representative lipid classes affected by lactate. Lactate selectively increased PE‐MUFAs, particularly PE‐OA‐containing species, whereas PC‐MUFAs were not significantly changed (Figure 5B,C). In contrast, arachidonic acid‐containing PE species were decreased after lactate treatment (Figure 5D). These findings suggest that lactate promotes PE remodeling toward a less peroxidation‐prone PE‐MUFA‐enriched lipid state.

Several researches has indicated that MUFAs, catalyzed by stearoyl‐CoA desaturase‐1 (SCD1), may effectively inhibit ferroptosis by substituting PUFAs in the lipid membrane and diminishing the accumulation of lipid ROS [34, 35]. Furthermore, in cells exhibiting predominant MBOAT2 activity, PE remodeling tends to shift the cellular composition toward a high PE‐MUFA level and a low PE‐PUFA level, thereby conferring resistance to ferroptosis [22]. In light of these findings, we proceeded to evaluate the expression levels of MBOAT2 and SCD1 using western blot analysis. Immunoblotting showed that MBOAT2 protein expression was higher in MES GSCs than in PN GSCs, whereas SCD1 expression was not significantly different between the two subtypes (Figure 5E,F). In WL1 and 2907 MES GSCs, lactate increased MBOAT2 protein expression, whereas LDHA knockdown or oxamate treatment attenuated lactate‐associated MBOAT2 induction. In contrast, SCD1 expression remained largely unchanged under these conditions (Figure 5G–I). Similarly, the levels of the canonical ferroptosis suppressors FSP1 and GPX4 were not substantially affected by lactate supplementation or LDHA depletion (Figure 5J–L). These data suggest that the LDHA/lactate axis regulates ferroptosis sensitivity mainly through MBOAT2‐associated lipid remodeling rather than through broad alteration of canonical GPX4‐ or FSP1‐dependent ferroptosis defense pathways.

To determine whether MBOAT2‐mediated lipid remodeling functionally contributes to ferroptosis resistance and radioresistance, we performed lipid rescue experiments using PE‐OA 18:1, a representative PE‐MUFA species increased after lactate treatment. MBOAT2 depletion increased lipid ROS accumulation and γ‐H2AX‐positive cells after irradiation, whereas PE‐OA 18:1 supplementation partially rescued these effects (Figure S4A–L). In contrast, PE‐FA 20:4 did not produce a comparable protective effect. These results indicate that PE‐MUFA remodeling, particularly PE‐OA 18:1 enrichment, contributes to the ferroptosis‐suppressive and radioprotective function of MBOAT2 in MES GSCs.

We next examined whether the enzymatic activity of MBOAT2 is required for its protective function. Before functional rescue experiments, we confirmed by immunoblotting that MBOAT2‐WT and the catalytically impaired MBOAT2‐H373A mutant were expressed at comparable levels in both WL1 and 2907 GSCs (Figure S5A,B). Thus, the differential effects of MBOAT2‐WT and MBOAT2‐H373A were not attributable to unequal protein expression. Under irradiation, wild‐type MBOAT2 reduced C11‐BODIPY lipid ROS accumulation, γ‐H2AX‐positive cells, and TUNEL‐positive cell death, whereas the H373A mutant failed to confer comparable protection, particularly under LDHA‐depleted conditions (Figure 5M–R). These findings indicate that MBOAT2 enzymatic activity is required to suppress ferroptosis‐associated lipid peroxidation and radiation‐induced damage in MES GSCs.

Collectively, these results support a model in which lactate‐induced MBOAT2 expression promotes PE‐MUFA remodeling, thereby limiting ferroptosis‐associated lipid peroxidation and contributing to radioresistance in MES GSCs.

### 3.6 LDHA Stabilizes MBOAT2 by Limiting Ubiquitin–Proteasome‐Mediated Degradation

**FIGURE 6:**
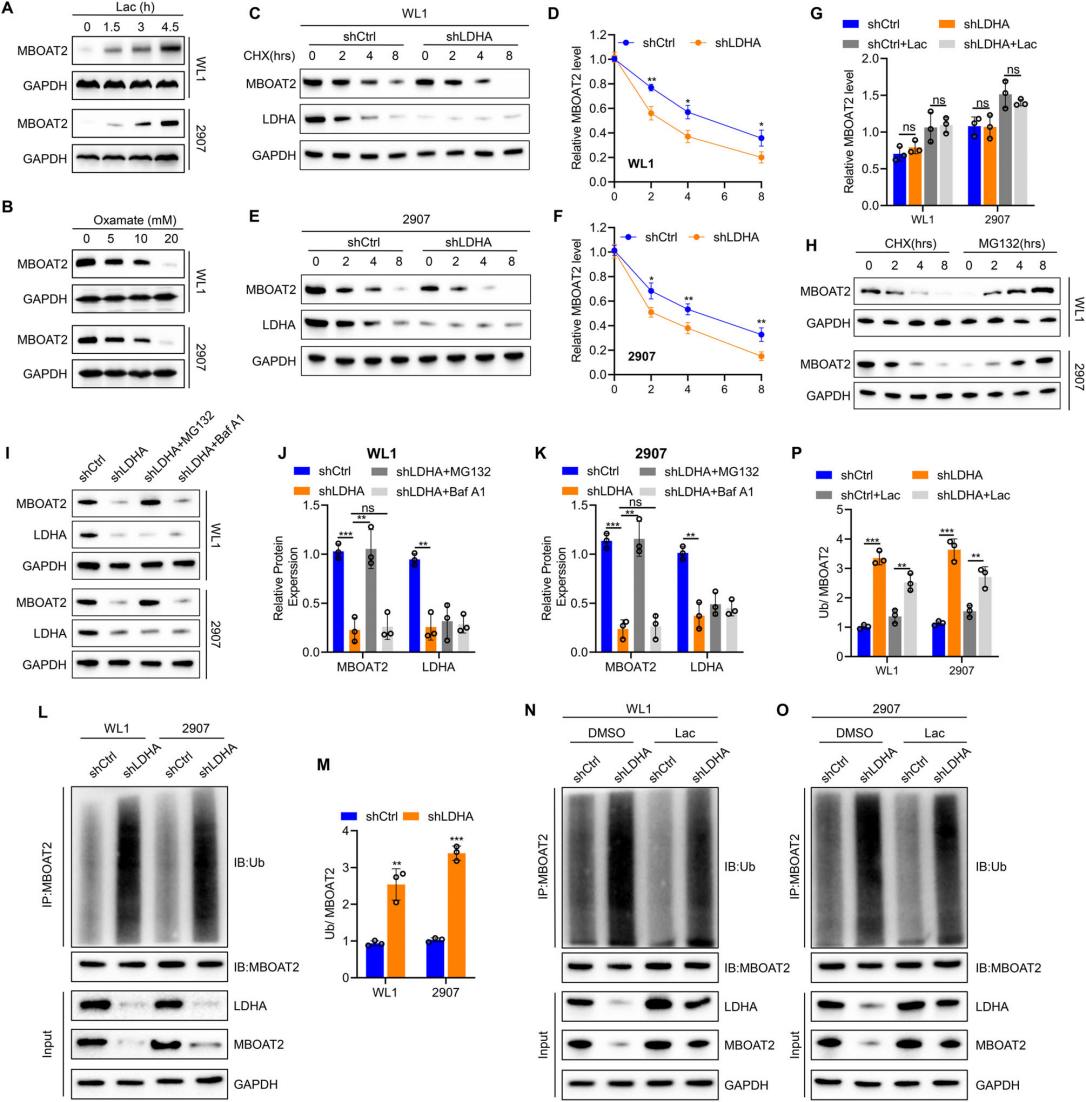
LDHA stabilizes MBOAT2 by limiting ubiquitin–proteasome‐mediated degradation. (A) Immunoblot analysis of MBOAT2 expression in WL1 and 2907 MES GSCs treated with lactate (12 mM) for the indicated times. GAPDH was used as the loading control. (B) Immunoblot analysis of MBOAT2 expression in WL1 and 2907 cells treated with oxamate (0, 5, 10, or 20 mM, 24 h). GAPDH was used as the loading control. (C–F) CHX chase assay showing MBOAT2 protein stability in control and LDHA‐depleted WL1 (C,D) and 2907 (E,F) cells. Cells were treated with CHX (100 µg/mL) and harvested at the indicated time points. MBOAT2 levels were normalized to GAPDH and expressed relative to the 0 h time point. (G) qRT‐PCR analysis of MBOAT2 mRNA levels in WL1 and 2907 cells with or without LDHA knockdown and lactate supplementation in the presence of actinomycin D (5 µg/mL, 6 h). (H) Immunoblot analysis of MBOAT2 stability after CHX chase in the presence or absence of MG132. Cells were pretreated with MG132 (20 µM, 1 h) where indicated, followed by CHX (100 µg/mL) treatment for the indicated time points in the continued presence or absence of MG132. (I–K) Immunoblot analysis (I) and quantification (J,K) of MBOAT2 expression in WL1 and 2907 cells transduced with shCtrl or shLDHA and treated with MG132 (20 µM, 8 h) or bafilomycin A1 (50 nM, 1 h) where indicated. (L,M) Endogenous MBOAT2 ubiquitination assay (L) and quantification (M) in WL1 and 2907 cells with or without LDHA knockdown. Cells were treated with MG132 (20 µM, 8 h) before harvest. MBOAT2 was immunoprecipitated, and ubiquitination was detected by immunoblotting. (N–P) Endogenous MBOAT2 ubiquitination assays in WL1 (N) and 2907 (O) cells under control or LDHA‐depleted conditions with or without lactate supplementation, with quantification shown in (P). Cells were treated with lactate (12 mM, 24 h) and MG132 (20 µM, 8 h) before harvest. For ubiquitination assays, ubiquitination signals were normalized to immunoprecipitated MBOAT2. For protein expression assays, MBOAT2 was normalized to GAPDH. Data are presented as mean ± SEM from *n* = 3 independent biological experiments. Statistical significance was determined by unpaired two‐tailed Student's t‐test or one‐way ANOVA followed by Tukey's multiple‐comparison test, as appropriate. *p < 0.05, **p < 0.01, ***p < 0.001; ns, not significant.

We next investigated how the LDHA/lactate axis regulates MBOAT2 protein abundance. Exogenous lactate increased MBOAT2 protein expression in a time‐dependent manner, whereas oxamate treatment reduced MBOAT2 levels in WL1 and 2907 MES GSCs (Figure 6A,B). To determine whether LDHA affects MBOAT2 protein stability, we performed cycloheximide chase assays. LDHA knockdown accelerated MBOAT2 degradation and shortened the half‐life of endogenous MBOAT2 in both GSC lines (Figure 6C–F), indicating that LDHA is required for maintaining MBOAT2 protein stability. To further confirm the causal role of lactate in LDHA‐mediated MBOAT2 stabilization, we performed CHX chase assays in LDHA‐depleted cells with exogenous lactate supplementation. Lactate partially restored MBOAT2 stability in shLDHA‐expressing WL1 and 2907 GSCs, as reflected by delayed MBOAT2 degradation compared with LDHA depletion alone (Figure S6A–D). These results support that LDHA maintains MBOAT2 protein stability, at least in part, through lactate production.

We then examined whether this regulation occurred at the transcriptional or post‐translational level. In the presence of actinomycin D, LDHA depletion or lactate supplementation did not significantly alter MBOAT2 mRNA levels (Figure 6G), suggesting that LDHA/lactate‐associated regulation of MBOAT2 is unlikely to be explained primarily by transcriptional induction. In contrast, MG132 attenuated CHX‐induced MBOAT2 degradation, and MG132, but not bafilomycin A1, restored MBOAT2 protein levels in LDHA‐depleted cells (Figure 6H–K). These findings suggest that LDHA depletion promotes proteasome‐dependent degradation of MBOAT2.

Consistently, endogenous ubiquitination assays showed that LDHA knockdown increased MBOAT2 ubiquitination, whereas lactate supplementation reduced MBOAT2 ubiquitination and partially restored MBOAT2 protein abundance under LDHA‐depleted conditions (Figure 6L–P). Together, these results indicate that the LDHA/lactate axis stabilizes MBOAT2 by limiting its ubiquitin–proteasome‐mediated degradation in MES GSCs.

### 3.7 LDHA‐Derived Lactate Promotes p300‐Dependent H3K18 Lactylation in MES GSCs

In our previous analysis of metabolic pathway enrichment, we observed a significant elevation in lactate content within MES GSCs. Given the extensive evidence from prior studies highlighting lactate's role as a precursor in lactylation regulation [36, 37, 38], we next examined whether lactate‐associated histone lactylation contributes to MES GSC radioresistance. Immunoblot analysis showed that histone‐associated pan‐lysine lactylation was higher in MES GSCs than in PN GSCs. Among multiple histone lactylation marks examined, H3K18la was prominently increased in MES GSCs and was therefore selected for further mechanistic investigation (Figure S7A–D). Consistently, p300 expression was also higher in MES GSCs than in PN GSCs, and p300 knockdown reduced H3K18la levels (Figure S7E–G).

**FIGURE 7:**
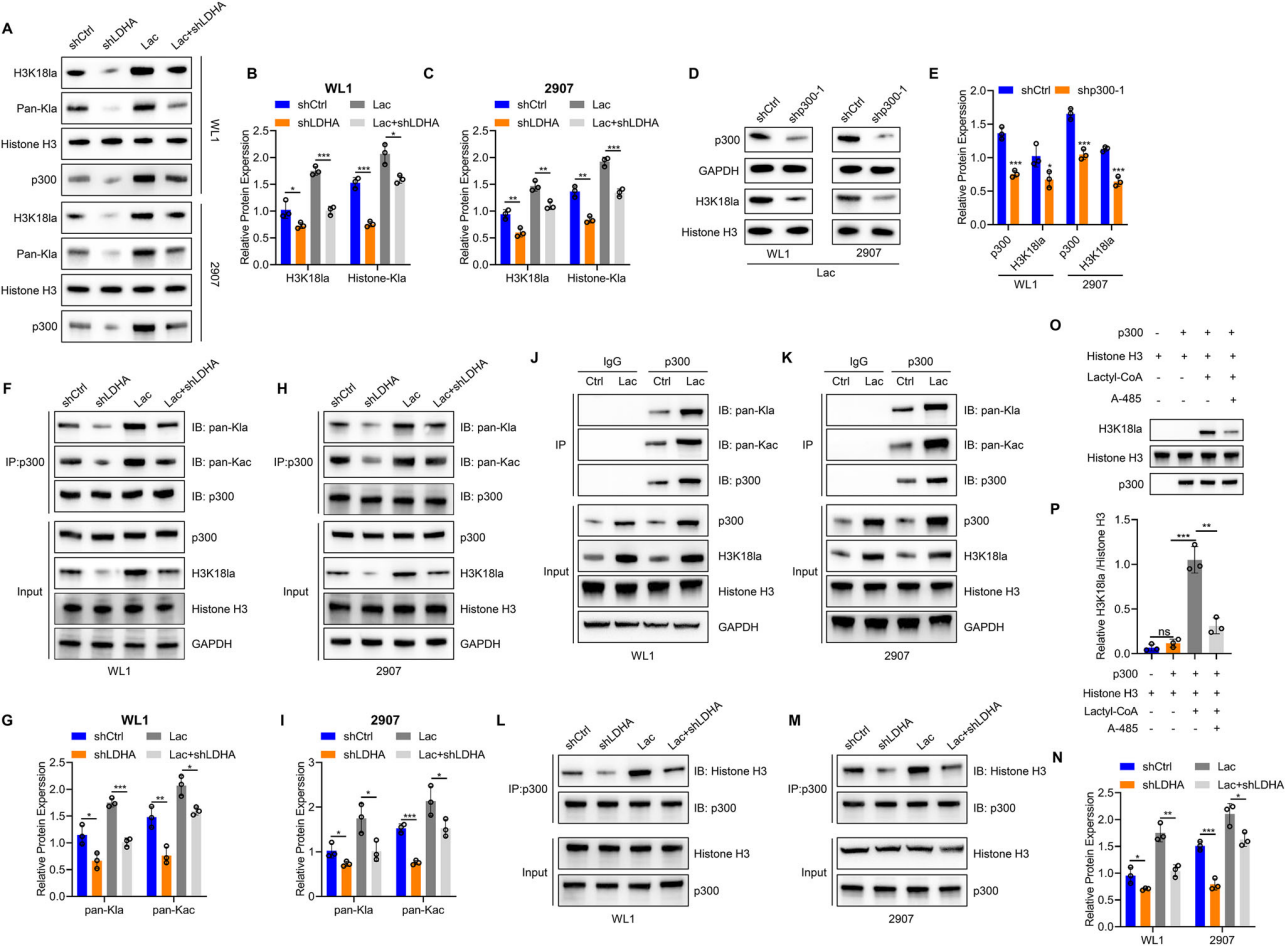
LDHA‐derived lactate promotes p300‐dependent H3K18 lactylation in MES GSCs. (A–C) Immunoblot analysis (A) and quantification (B,C) of H3K18la and histone‐associated pan‐Kla in WL1 and 2907 MES GSCs with or without LDHA knockdown and lactate supplementation. Lactate was used at 12 mM for 24 h. H3K18la and pan‐Kla signals were normalized to total Histone H3. (D, E) Immunoblot analysis (D) and quantification (E) of p300 and H3K18la in WL1 and 2907 cells after p300 knockdown. p300 was normalized to GAPDH, and H3K18la was normalized to total Histone H3. (F–I) Immunoprecipitation analysis of endogenous p300 lactylation and acetylation in WL1 (F,G) and 2907 (H,I) cells under the indicated conditions. p300 was immunoprecipitated and immunoblotted with pan‐Kla, pan‐Kac, and p300 antibodies. Lactate was used at 12 mM for 24 h. Pan‐Kla and pan‐Kac signals were normalized to immunoprecipitated p300. (J,K) IgG control for p300 immunoprecipitation in WL1 and 2907 cells. Normal IgG or anti‐p300 antibody was used for immunoprecipitation, followed by immunoblotting with pan‐Kla, pan‐Kac, and p300 antibodies. (L–N) Co‐immunoprecipitation analysis of the interaction between endogenous p300 and histone H3 in WL1 (L) and 2907 (M) cells, with quantification shown in (N). Histone H3 signals in p300 immunoprecipitates were normalized to immunoprecipitated p300. (O, P) In vitro lactylation assay (O) and quantification (P) using recombinant p300, recombinant histone H3, and lactyl‐CoA. The p300/CBP inhibitor A‐485 was added where indicated. H3K18la signals were normalized to total Histone H3. Data are presented as mean ± SEM from *n* = 3 independent biological experiments, unless otherwise indicated. Statistical significance was determined by unpaired two‐tailed Student's t‐test or one‐way ANOVA followed by Tukey's multiple‐comparison test, as appropriate. *p < 0.05, **p < 0.01, ***p < 0.001; ns, not significant.

We then tested whether the LDHA/lactate axis directly regulates H3K18la in MES GSCs. LDHA depletion reduced H3K18la and histone‐associated pan‐Kla levels in WL1 and 2907 cells, whereas exogenous lactate restored these lactylation signals (Figure 7A–C). In addition, p300 knockdown markedly reduced lactate‐induced H3K18la, supporting a requirement for p300 in lactate‐associated H3K18 lactylation (Figure 7D,E).

To further address whether lactate regulates p300‐associated post‐translational modification, endogenous p300 was immunoprecipitated and examined using pan‐Kla and pan‐Kac antibodies. Lactate increased p300‐associated lactylation and acetylation signals, whereas LDHA depletion reduced these modifications; lactate supplementation partially restored them under LDHA‐depleted conditions (Figure 7F–I). IgG immunoprecipitation showed minimal background signals, confirming the specificity of p300 pull‐down (Figure 7J,K). Co‐immunoprecipitation further showed that lactate enhanced the association between p300 and histone H3, whereas LDHA knockdown weakened this interaction (Figure 7L–N).

Finally, an in vitro lactylation assay using recombinant p300, recombinant histone H3, and lactyl‐CoA showed that p300 induced H3K18 lactylation in a lactyl‐CoA‐dependent manner. This reaction was attenuated by the p300/CBP inhibitor A‐485 (Figure 7O,P). Together, these results provide cellular and biochemical evidence that LDHA‐derived lactate promotes p300‐dependent H3K18 lactylation in MES GSCs.

### 3.8 LDHA‐mediated H3K18 Lactylation Enhances MBOAT2 Expression by Promoting USP30 Transcription

**FIGURE 8:**
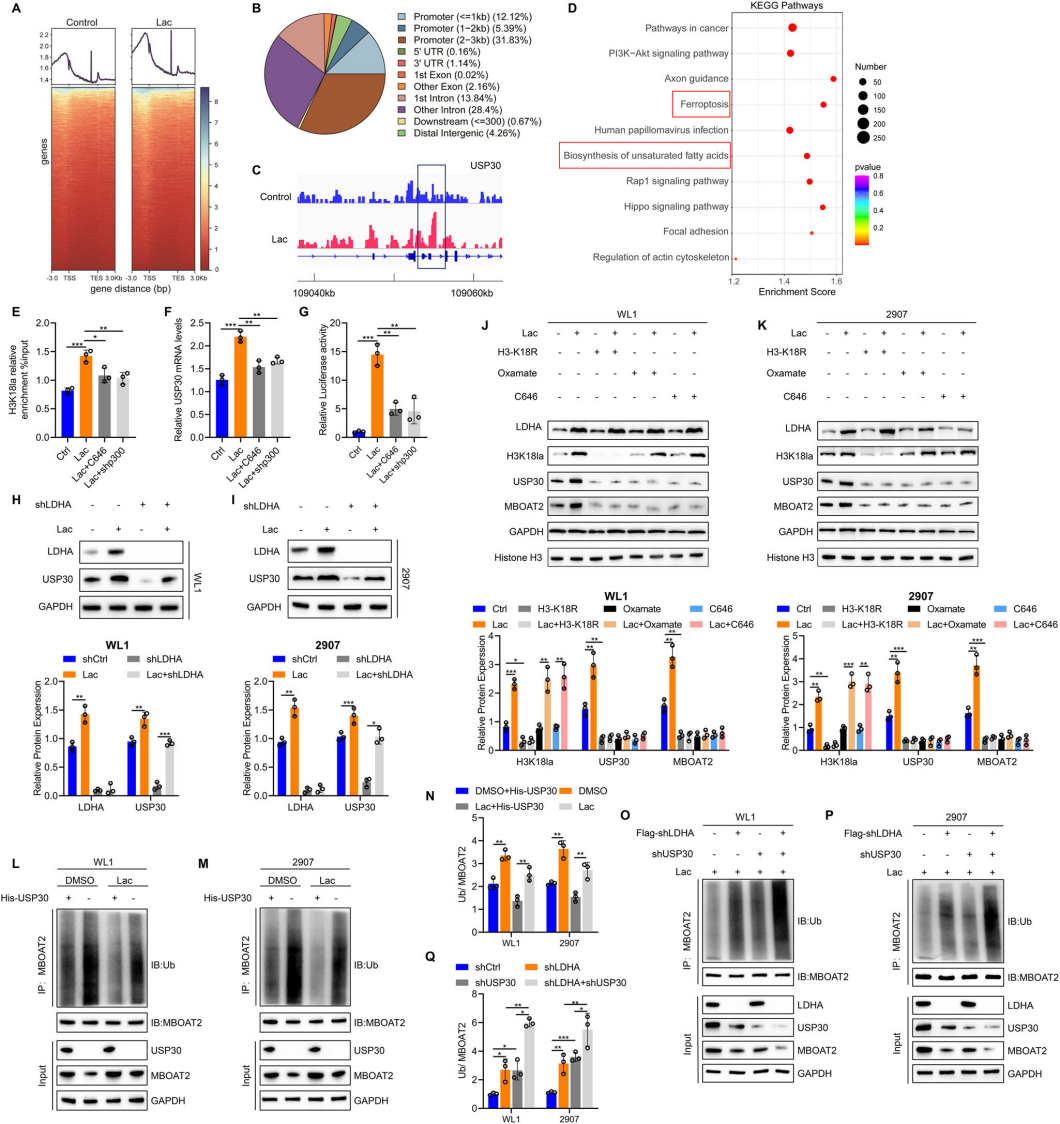
LDHA‐mediated H3K18 lactylation enhances MBOAT2 expression by promoting USP30 transcription. (A) Heatmap showing H3K18la ChIP‐seq signal density around transcription start sites in WL1 MES GSCs treated with or without lactate (12 mM, 24 h). (B) Genomic distribution of H3K18la peaks in lactate‐treated WL1 cells. (C) IGV tracks showing H3K18la enrichment at the USP30 promoter region in control and lactate‐treated WL1 cells. (D) KEGG pathway enrichment analysis of H3K18la‐associated genes in lactate‐treated WL1 cells. (E) ChIP‐qPCR analysis of H3K18la enrichment at the USP30 promoter under the indicated conditions. Lactate was used at 12 mM for 24 h. C646 was used at 25 µM for 24 h where indicated. Data are shown as mean ± SEM from *n* = 3 independent biological experiments. (F) qRT‐PCR analysis of USP30 mRNA expression in WL1 cells under the indicated conditions. GAPDH was used as the internal control. (G) USP30 promoter luciferase reporter assay under the indicated conditions. Firefly luciferase activity was normalized to Renilla luciferase activity. (H,I) Immunoblot analysis and quantification of LDHA and USP30 expression in LDHA‐depleted WL1 (H) and 2907 (I) cells with or without lactate supplementation. Lactate was used at 12 mM for 24 h. GAPDH was used as the loading control. (J,K) Immunoblot analysis and quantification of LDHA, H3K18la, USP30, and MBOAT2 in WL1 (J) and 2907 (K) cells under the indicated treatments. Lactate was used at 12 mM for 24 h, oxamate at 20 mM for 24 h, and C646 at 25 µM, 24 h. H3K18la was normalized to Histone H3, whereas LDHA, USP30, and MBOAT2 were normalized to GAPDH. (L, M) Endogenous MBOAT2 ubiquitination assays in WL1 (L) and 2907 (M) cells with or without His‐USP30 overexpression and lactate treatment. Cells were treated with lactate (12 mM, 24 h) and MG132 (20 µM, 8 h) before harvest. MBOAT2 was immunoprecipitated and ubiquitination was detected by immunoblotting. (N) Quantification of ubiquitinated MBOAT2 levels shown in (L,M). Ubiquitination signals were normalized to immunoprecipitated MBOAT2. (O,P) Endogenous MBOAT2 ubiquitination assays in LDHA‐depleted WL1 (O) and 2907 (P) cells with or without USP30 knockdown and lactate supplementation. Cells were treated with lactate (12 mM, 24 h) and MG132 (20 µM, 8 h) before harvest. (Q) Quantification of ubiquitinated MBOAT2 levels shown in (O,P). Ubiquitination signals were normalized to immunoprecipitated MBOAT2. Data are presented as mean ± SEM from n = 3 independent biological experiments unless otherwise indicated. Statistical significance was determined by unpaired two‐tailed Student's t‐test or one‐way ANOVA followed by Tukey's multiple‐comparison test, as appropriate. *p < 0.05, **p < 0.01, ***p < 0.001; ns, not significant.

To identify downstream genes regulated by lactate‐induced H3K18la, we performed H3K18la ChIP‐seq in WL1 MES GSCs treated with or without lactate. H3K18la signals were broadly distributed around transcriptional regulatory regions, with a substantial fraction of peaks located in promoter and upstream regions (Figure 8A,B). IGV visualization revealed a lactate‐enhanced H3K18la peak at the USP30 promoter region (Figure 8C). KEGG pathway analysis of H3K18la‐associated genes showed enrichment of pathways related to ferroptosis and biosynthesis of unsaturated fatty acids (Figure 8D), suggesting a potential link between lactate‐induced histone lactylation and ferroptosis‐regulatory lipid metabolism.

Given that USP30 is a deubiquitinating enzyme potentially involved in MBOAT2 stabilization, we focused on USP30 as a candidate H3K18la‐regulated gene. ChIP‐qPCR confirmed that lactate increased H3K18la enrichment at the USP30 promoter, whereas p300 inhibition or p300 knockdown attenuated this enrichment (Figure 8E). Consistently, lactate increased USP30 mRNA expression and USP30 promoter luciferase activity, both of which were reduced by p300 inhibition or p300 knockdown (Figure 8F,G). These data support that lactate promotes USP30 transcription through a p300/H3K18la‐dependent mechanism.

We next examined whether LDHA‐derived lactate regulates USP30 expression in MES GSCs. LDHA knockdown reduced USP30 protein expression, whereas exogenous lactate partially restored USP30 levels in both WL1 and 2907 cells (Figure 8H,I). Furthermore, lactate increased H3K18la, USP30, and MBOAT2 expression, whereas H3K18R expression, oxamate treatment, or p300 inhibition by C646 suppressed lactate‐induced USP30 and MBOAT2 upregulation (Figure 8J,K). These results suggest that the LDHA/lactate–H3K18la axis promotes MBOAT2 expression through USP30 transcriptional induction. Finally, we investigated whether USP30 regulates MBOAT2 ubiquitination. His‐USP30 overexpression reduced MBOAT2 ubiquitination and increased MBOAT2 abundance, and this effect was further supported under lactate‐treated conditions (Figure 8L–N). Conversely, USP30 knockdown enhanced MBOAT2 ubiquitination and impaired the ability of lactate to restore MBOAT2 stability under LDHA‐depleted conditions (Figure 8O–Q).

To determine whether USP30 directly interacts with MBOAT2, we first performed reciprocal endogenous Co‐IP assays. MBOAT2 immunoprecipitation pulled down USP30, and USP30 immunoprecipitation reciprocally detected MBOAT2 in both WL1 and 2907 cells (Figure S8A). GST pull‐down assays further confirmed a direct interaction between USP30 and MBOAT2 (Figure S8B). We then examined whether the deubiquitinase activity of USP30 is required for MBOAT2 stabilization. Compared with the catalytically inactive USP30‐C77A mutant, wild‐type USP30 more effectively increased MBOAT2 protein abundance and reduced MBOAT2 ubiquitination (Figure S8C,D), indicating that USP30 stabilizes MBOAT2 in a catalytic activity‐dependent manner. In addition, mutagenesis of candidate lysine residues identified MBOAT2 Lys48 as a major functional ubiquitination site, as the MBOAT2‐Lys48R mutant showed markedly reduced ubiquitination and was less sensitive to USP30 knockdown‐induced destabilization (Figure S8E,F).

Together, these findings indicate that LDHA‐derived lactate promotes p300‐dependent H3K18la at the USP30 promoter, thereby increasing USP30 transcription; USP30 then physically interacts with MBOAT2 and stabilizes MBOAT2, at least in part, by limiting MBOAT2 Lys48 ubiquitination.

### 3.9 Targeting the LDHA–USP30–MBOAT2 Axis Enhances Radiosensitivity by Promoting Ferroptosis In Vitro and In Vivo

**FIGURE 9:**
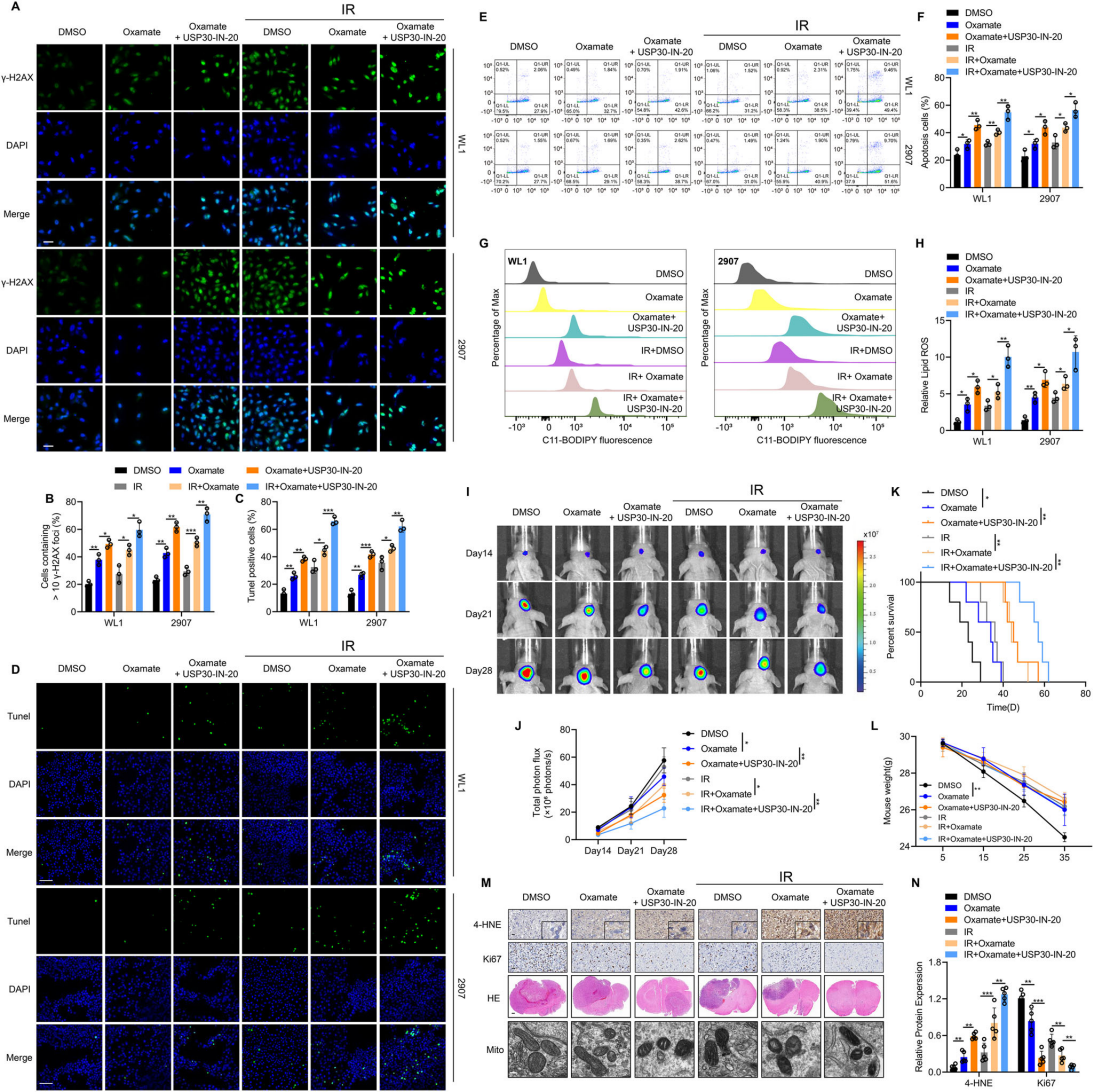
Targeting the LDHA–USP30–MBOAT2 axis enhances radiosensitivity by promoting ferroptosis in vitro and in vivo. (A,B) Representative γ‐H2AX immunofluorescence images (A) and quantification of γ‐H2AX‐positive cells (B) in WL1 and 2907 MES GSCs under the indicated treatments. Cells were treated with oxamate (20 mM, 24 h) and/or USP30‐IN‐20 (5 µM, 24 h), exposed to 4 Gy irradiation, and fixed at 12 h post‐IR. Scale bar, 50 µm. (C,D) Quantification of TUNEL‐positive cells (C) and representative TUNEL staining images (D) under the indicated treatments. Cells were analyzed at 24 h post‐IR. Scale bar, 100 µm. (E,F) Representative Annexin V/PI flow cytometry plots (E) and quantification of apoptotic cells (F) under the indicated treatments. (G,H) Representative C11‐BODIPY flow cytometry histograms (G) and quantification of lipid ROS levels (H). Cells were analyzed at 12 h post‐IR, and lipid ROS was detected using BODIPY 581/591 C11 (1.5 µM, 30 min). (I,J) Representative IVIS bioluminescence images (I) and quantification of total photon flux (J) in intracranial WL1 GSC xenografts. Oxamate was administered intraperitoneally at 300 mg/kg once daily, and USP30‐IN‐20 was administered by oral gavage at 10 mg/kg once daily. Local cranial irradiation was delivered as 2.5 Gy per fraction for four consecutive days. *n* = 5 male nude mice per group. (K) Kaplan–Meier survival analysis of mice bearing intracranial WL1 GSC xenografts. *n* = 5 male nude mice per group. (L) Body weight monitoring of tumor‐bearing mice during treatment. (M,N) Representative IHC staining for 4‐HNE and Ki67, H&E staining, TEM images (M), and quantification of 4‐HNE and Ki67 staining (N) in xenograft tumors. IHC staining was quantified based on staining intensity and the proportion of positive tumor cells. Scale bars, 50 µm for IHC, 1 mm for H&E, and 1 µm for TEM. Data are presented as mean ± SEM from *n* = 3 independent biological experiments for in vitro assays unless otherwise indicated. Statistical significance was determined by one‐way ANOVA, two‐way ANOVA, or log‐rank test as appropriate. *p < 0.05, **p < 0.01, ***p < 0.001.

We next investigated whether combined targeting of lactate production and USP30‐mediated MBOAT2 stabilization could enhance the radiosensitivity of MES GSCs. In WL1 and 2907 cells, oxamate treatment increased the proportion of γ‐H2AX‐positive cells after irradiation, and this effect was further enhanced by the USP30 inhibitor USP30‐IN‐20 (Figure 9A,B). Consistently, combined oxamate and USP30‐IN‐20 treatment increased TUNEL‐positive cells and Annexin V‐positive apoptotic cells following irradiation (Figure 9C–F). C11‐BODIPY flow cytometry further showed that oxamate increased lipid ROS accumulation, which was further elevated by USP30‐IN‐20, particularly under irradiated conditions (Figure 9G,H). These data suggest that simultaneous inhibition of LDHA activity and USP30‐mediated MBOAT2 stabilization promotes ferroptosis‐associated lipid peroxidation and enhances radiation‐induced DNA damage and cell death in MES GSCs.

We further evaluated this therapeutic strategy in an intracranial WL1 GSC xenograft model. Combined oxamate, USP30‐IN‐20, and irradiation markedly suppressed tumor growth, as assessed by IVIS bioluminescence imaging and total photon flux quantification, and significantly prolonged survival compared with irradiation alone or dual‐treatment groups (Figure 9I–K). Body weight monitoring showed no obvious additional treatment‐associated weight loss in the combination group (Figure 9L). Histological analysis further showed increased 4‐HNE staining and reduced Ki67 staining after combined treatment, indicating enhanced lipid peroxidation and reduced tumor proliferation (Figure 9M,N). Transmission electron microscopy revealed mitochondrial morphological changes consistent with ferroptosis, including shrunken mitochondria and disrupted mitochondrial membranes, in tumors receiving the combination treatment (Figure 9M). Together, these findings indicate that targeting the LDHA–USP30–MBOAT2 axis enhances the antitumor efficacy of radiotherapy by promoting ferroptosis‐associated lipid peroxidation in MES GSCs.

### 3.10 Clinical Relevance of the LDHA–H3K18la–USP30–MBOAT2 Axis in GBM

**FIGURE 10:**
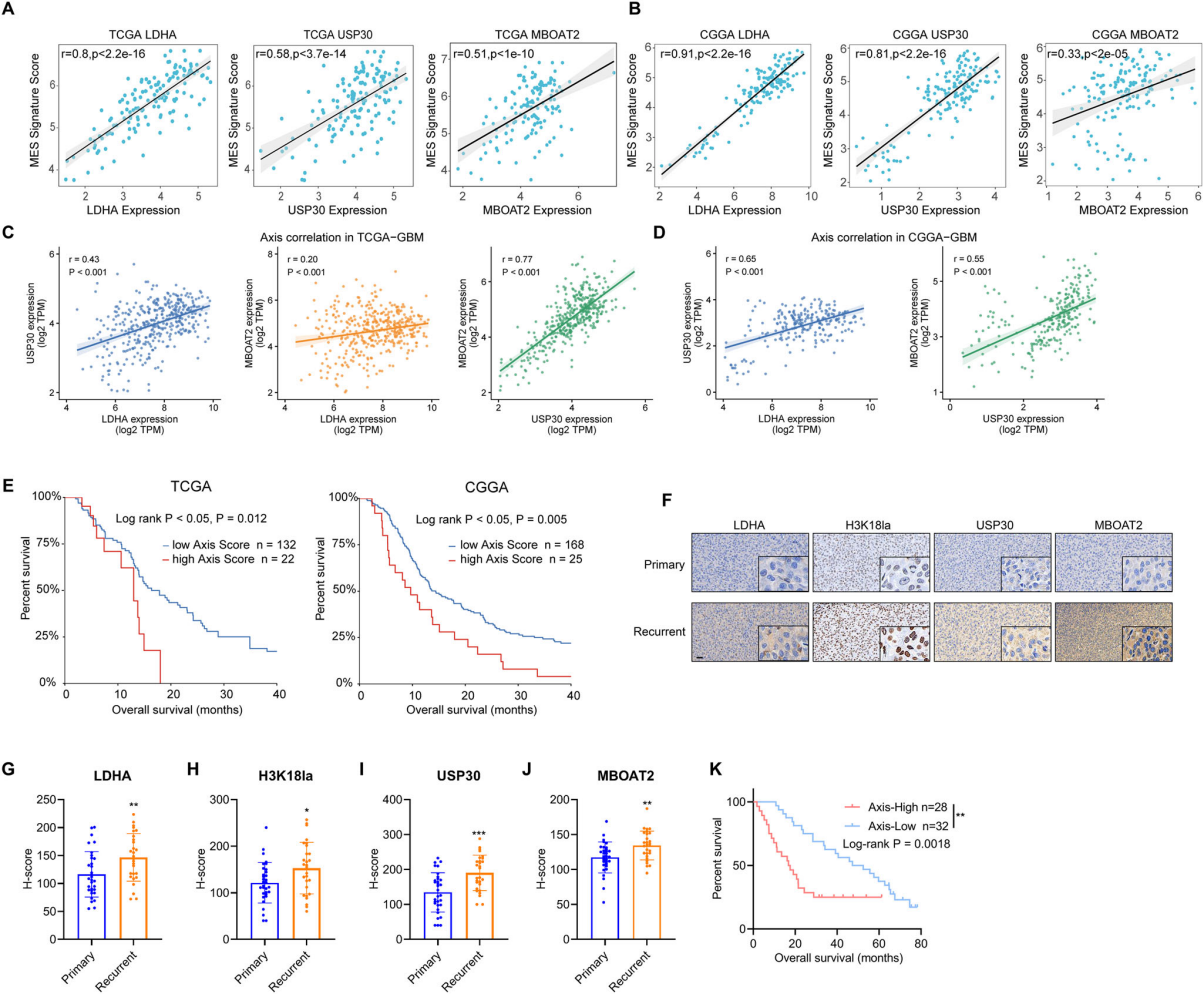
Clinical relevance of the LDHA–H3K18la–USP30–MBOAT2 axis in GBM. (A,B) Correlation analysis between MES signature score and LDHA, USP30, or MBOAT2 expression in TCGA‐GBM (A) and CGGA‐GBM (B) cohorts. The MES signature score was calculated as the average expression of Verhaak MES signature genes. Spearman correlation coefficients and *p* values are shown. (C,D) Pairwise correlation analysis of LDHA, USP30, and MBOAT2 expression in TCGA‐GBM (C) and CGGA‐GBM (D) cohorts. Gene expression values are shown as log2‐transformed TPM or normalized expression values, as appropriate. Spearman correlation coefficients and *p* values are indicated. (E) Kaplan–Meier analysis of overall survival in radiotherapy‐treated GBM patients from TCGA and CGGA cohorts. Patients were stratified into axis‐high and axis‐low groups using the optimal cutpoint of the LDHA–USP30–MBOAT2 transcriptomic axis score. Statistical significance was determined by log‐rank test. (F) Representative IHC staining images of LDHA, H3K18la, USP30, and MBOAT2 in primary and recurrent GBM specimens. LDHA, USP30, and MBOAT2 were mainly scored based on cytoplasmic staining, whereas H3K18la was scored based on nuclear staining. Scale bar, 50 µm. (G–J) Quantification of LDHA (G), H3K18la (H), USP30 (I), and MBOAT2 (J) staining in primary and recurrent GBM specimens using H‐scores. Primary, *n* = 32; recurrent, *n* = 28. Data are shown as mean ± SD. Statistical significance was determined by Mann–Whitney U test. (K) Kaplan–Meier analysis of overall survival in the in‐house GBM cohort stratified by the LDHA–H3K18la–USP30–MBOAT2 protein‐axis score. Patients were divided into protein‐axis‐high and protein‐axis‐low groups using the median protein‐axis score. Axis‐high, *n* = 28; axis‐low, *n* = 32. Statistical significance was determined by log‐rank test. *p < 0.05, **p < 0.01, ***p < 0.001.

To assess the clinical relevance of the LDHA–H3K18la–USP30–MBOAT2 axis, we first analyzed TCGA‐GBM and CGGA‐GBM transcriptomic datasets. Because H3K18la is a histone post‐translational modification and cannot be directly inferred from RNA‐seq data, public dataset analyses focused on the transcriptomic components LDHA, USP30, and MBOAT2. The MES signature score was calculated as the average expression of Verhaak MES signature genes. LDHA, USP30, and MBOAT2 showed positive associations with MES signature scores in both TCGA and CGGA cohorts, although the strength of correlation varied across genes and datasets (Figure 10A,B).

We next examined the pairwise relationships among LDHA, USP30, and MBOAT2. Correlation analysis showed positive associations among axis components, with a particularly consistent correlation between USP30 and MBOAT2 in TCGA and CGGA cohorts (Figure 10C,D). We then evaluated whether this transcriptomic axis was associated with radiotherapy‐related prognosis. In radiotherapy‐treated GBM patients from TCGA and CGGA cohorts, patients with high LDHA–USP30–MBOAT2 axis scores, stratified using the optimal cutpoint, exhibited significantly shorter overall survival than those with low axis scores (Figure 10E).

To further validate this axis at the protein level, we performed IHC analysis of LDHA, H3K18la, USP30, and MBOAT2 in an in‐house GBM cohort containing primary and recurrent tumor specimens. Compared with primary tumors, recurrent tumors showed increased staining of LDHA, H3K18la, USP30, and MBOAT2 (Figure 10F–J). Finally, patients with high LDHA–H3K18la–USP30–MBOAT2 protein‐axis scores showed significantly poorer overall survival than those with low protein‐axis scores (Figure 10K). Collectively, these clinical analyses support the relevance of the LDHA–H3K18la–USP30–MBOAT2 axis in GBM recurrence and radiotherapy‐associated poor prognosis.

## 4 Discussion

Radiotherapy continues to be a fundamental component in the multidisciplinary treatment of glioma, especially for high‐grade tumors [39, 40]. Despite advances in precision techniques such as intensity‐modulated radiation therapy and proton therapy, therapeutic efficacy remains constrained by intrinsic radioresistance and inevitable recurrence. This resistance is shaped by multiple factors, including tumor hypoxia, metabolic adaptation, and the persistence of glioma stem cells (GSCs) [9, 10, 12]. Among GSC subtypes, mesenchymal (MES) GSCs are particularly associated with tumor recurrence, aggressive progression, and poor therapeutic response. In this study, we identify a metabolic–epigenetic–lipid remodeling mechanism by which MES GSCs exploit LDHA‐mediated lactate production to suppress ferroptosis and promote radioresistance.

Tumor metabolism has traditionally been regarded as a downstream consequence of oncogenic signaling; however, accumulating evidence suggests that metabolic dysregulation can actively drive tumor initiation, progression, and cellular state transitions [18, 41]. Metabolites generated from aberrant metabolic pathways can serve as cofactors or substrates for chromatin‐modifying enzymes, thereby reshaping epigenetic landscapes and transcriptional programs [42]. Extensive research has identified GSCs as principal contributors to glioblastoma propagation and recurrence [43, 44]. Consistent with this concept, we found that MES GSCs exhibit a distinct metabolic profile characterized by increased glycolytic activity, elevated LDHA expression, and enhanced lactate production. Lactate, once regarded merely as an inert byproduct of glycolysis, is now recognized as a key signaling metabolite with diverse roles in tumor biology [16, 45, 46].

Our findings further support this concept by showing that lactate decreases ferroptosis sensitivity, whereas LDHA inhibition enhances lipid ROS accumulation and ferroptotic vulnerability in MES GSCs. Our study first establishes a functional association between ferroptosis and MES‐state maintenance. Ferroptosis induction reduced MES‐associated markers and transcriptional regulators, including CD44, ALDH1A3, C/EBPβ, TAZ, and phosphorylated STAT3, and markedly impaired tumorsphere‐forming capacity. These effects were rescued by Ferrostatin‐1 but not by inhibitors of apoptosis or necroptosis, supporting a ferroptosis‐associated mechanism. Functionally, ferroptosis activation enhanced radiosensitivity, reduced clonogenic survival, prolonged γ‐H2AX foci persistence after irradiation, and increased post‐irradiation cell death. These observations suggest that ferroptosis sensitivity is closely linked to MES phenotype maintenance and radiotherapy response in GSCs. We further expand the understanding of lactate biology in glioblastoma by showing that LDHA‐derived lactate promotes histone lactylation in MES GSCs. Recent studies have implicated H3K18la in therapy resistance in other tumor types. H3K18la‐driven transcription factors YBX1 and YY1 have been reported to contribute to cisplatin resistance in bladder cancer cells [47], while H3K18la enhances resistance to bevacizumab in colorectal cancer by promoting RUBCNL expression [48]. In our study, MES GSCs displayed higher histone‐associated lactylation than PN GSCs, with H3K18la emerging as a prominent lactate‐responsive mark. Mechanistically, lactate‐induced H3K18la was dependent, at least in part, on p300. p300 knockdown suppressed lactate‐induced H3K18la, while p300 immunoprecipitation, p300–histone H3 interaction assays, and in vitro lactylation assays provided additional cellular and biochemical evidence supporting p300‐dependent H3K18 lactylation. Nevertheless, although lactate increased p300‐associated lactylation and acetylation signals, the specific modification sites on p300 and their direct contribution to p300 catalytic activity remain to be further defined.

A central finding of this work is that USP30 acts as a downstream transcriptional target of lactate‐induced H3K18la. H3K18la ChIP‐seq and ChIP‐qPCR revealed lactate‐enhanced H3K18la enrichment at the USP30 promoter. Consistently, lactate increased USP30 mRNA expression and USP30 promoter activity, whereas disruption of p300/H3K18la signaling attenuated these effects. USP30 subsequently stabilized MBOAT2 by reducing its ubiquitination. Thus, USP30 serves as a mechanistic bridge connecting lactate‐driven epigenetic regulation to post‐translational control of lipid remodeling.

MBOAT2 emerged as a key effector of this anti‐ferroptotic program. Lipidomic analysis showed that lactate shifted the membrane lipid profile toward monounsaturated fatty acid‐containing phosphatidylethanolamines, particularly PE‐MUFA species, while reducing arachidonic acid‐containing PE species that are more prone to peroxidation. MBOAT2 expression was elevated in MES GSCs and induced by lactate, whereas LDHA knockdown or oxamate treatment reduced MBOAT2 expression. Importantly, wild‐type MBOAT2, but not the catalytically impaired H373A mutant, reduced lipid ROS accumulation, γ‐H2AX‐positive cells, and TUNEL‐positive cell death after irradiation. These findings indicate that MBOAT2 enzymatic activity is required for suppressing ferroptosis‐associated lipid peroxidation and radiation‐induced damage. Therefore, lactate‐induced MBOAT2 stabilization provides MES GSCs with a lipid remodeling‐based defense mechanism against ferroptosis.

Radiotherapy resistance is an intrinsic property of GSCs and involves multiple mechanisms, including upregulation of drug efflux pumps, superior DNA repair capacity, and enhanced protection against ROS [17, 49]. Our data suggest that the LDHA–H3K18la–USP30–MBOAT2 axis represents an additional adaptive mechanism by which MES GSCs tolerate radiotherapy‐induced stress. Oxamate‐mediated LDHA inhibition increased radiation‐induced DNA damage persistence, lipid ROS accumulation, and cell death in MES GSCs, and these effects were further strengthened by the USP30 inhibitor USP30‐IN‐20. In intracranial xenograft models, combined targeting of LDHA and USP30 enhanced the antitumor efficacy of radiotherapy, suppressed tumor growth, increased 4‐HNE staining, reduced Ki67 expression, and prolonged survival. Body weight monitoring showed no obvious additional treatment‐associated weight loss under the tested conditions, although more comprehensive toxicity and pharmacokinetic analyses are required before clinical translation. However, further pharmacokinetic, toxicity, and blood–brain barrier penetration studies are needed before this therapeutic strategy can be considered for clinical translation.

Clinical analyses further supported the relevance of this pathway. In TCGA and CGGA cohorts, LDHA, USP30, and MBOAT2 were positively associated with MES signature scores, although the strength of correlation varied across genes and datasets. A high LDHA–USP30–MBOAT2 transcriptomic axis score was associated with poorer overall survival in radiotherapy‐treated GBM patients. Because H3K18la cannot be inferred directly from transcriptomic datasets, we further evaluated this axis at the protein level. Recurrent GBM specimens showed increased LDHA, H3K18la, USP30, and MBOAT2 expression compared with primary tumors, and a high protein‐axis score predicted worse survival. These findings support the clinical relevance of the LDHA–H3K18la–USP30–MBOAT2 axis in recurrent and therapy‐resistant GBM.

Several limitations should be acknowledged. First, TCGA and CGGA are bulk tumor datasets, and GSC‐specific regulatory programs may be partially masked by tumor heterogeneity and non‐tumor cell populations. Second, lactate is a multifunctional metabolite and may promote radioresistance through ferroptosis‐independent mechanisms, including chromatin remodeling, metabolic adaptation, immune regulation, and microenvironmental remodeling. Third, although our data support p300‐dependent H3K18 lactylation, the precise mechanism by which lactate regulates p300 activity requires further investigation. Finally, the in vivo experiments were performed in immunodeficient mouse models, which do not fully recapitulate immune–metabolic interactions in GBM.

In conclusion, our study elucidates a metabolic–epigenetic–lipid remodeling pathway underlying radioresistance in MES GSCs. We propose that elevated LDHA activity and lactate production promote p300‐dependent H3K18 lactylation, transcriptionally activate USP30, stabilize MBOAT2, and remodel membrane phospholipids toward a ferroptosis‐resistant state. Disrupting this pathway enhances ferroptosis‐associated lipid peroxidation and improves radiotherapy response, highlighting the LDHA–H3K18la–USP30–MBOAT2 axis as a potential therapeutic vulnerability for overcoming GBM radioresistance.

## 5 Conclusion

In conclusion, our study identifies a metabolic–epigenetic–lipid remodeling axis through which LDHA‐derived lactate promotes p300‐dependent H3K18 lactylation, activates USP30 transcription, stabilizes MBOAT2, and remodels membrane phospholipids toward a ferroptosis‐resistant state. Targeting this LDHA–H3K18la–USP30–MBOAT2 axis enhances ferroptosis‐associated lipid peroxidation and radiosensitivity in MES GSCs, providing a potential therapeutic strategy for overcoming GBM radioresistance.

## Author Contributions

Z.M., L.X., and J.X.C. designed and interpreted experiments. Z.M., W.G., Y.N.Z., W.Z.C., and J.N.L. performed experiments. Y.M.R., R.L., H.X.W., and C.C. analyzed the data. Z.M. and Z.Y.B. wrote the manuscript. C.F.L. and K.J. made contributions to the revision. All authors critically read the manuscript.

## Funding

This study was supported by grant from National Natural Science Foundation of China (82303915).

## Ethics Statement

The procedures were approved by the Animal Management Rule of the Chinese Ministry of Health (documentation 55, 2001) and the Nanjing Medical University Animal Experimental Ethics Committee. All the patients had signed informed consent and were enrolled according to the institutional protocols by the Ethics Committee of Changhai Hospital, Naval Medical University.

## Conflicts of Interest

The authors declare no conflicts of interest.

## Supporting information




**Supporting File 1**: advs77044‐sup‐0001‐SuppMat.docx.

**Supporting File 2**: advs77044‐sup‐0002‐FigureS1–S9.zip.

## Data Availability

The data that support the findings of this study are available on request from the corresponding author. The data are not publicly available due to privacy or ethical restrictions.
