# Supplementary material for: Metabolic Reprogramming in Glioblastoma Stem Cells Promotes Radiation Resistance Through a H3K18la/USP30/MBOAT2 Axis
Source: Adv Sci (Weinh). 2026 Aug 12;13(61):e77044. doi: 10.1002/advs.77044 (PMC13464552; doi:10.1002/advs.77044)
Supplement: Supplementary file 2 [file ADVS-13-e77044-s002.docx]

**Supplementary materials**

**Figure S1. Flow cytometry and functional validation of MES and PN GSC phenotypes.**
**(A)** Flow cytometry analysis of CD44 expression in WL1 and 2907 MES GSCs and CD133 expression in MGG8 and PN35 PN GSCs. Isotype controls are shown in black. **(B)** Flow cytometry analysis of CD44/ALDH1-positive populations in WL1 and 2907 MES GSCs and CD133/CD15-positive populations in MGG8 and PN35 PN GSCs. These data provide additional validation of subtype-associated surface marker profiles in MES and PN GSCs. **(C)** Representative tumorsphere images of CD44-negative and CD44-positive populations isolated from WL1, 2907, MGG8, and PN35 GSCs. Scale bar, 25 μm.

**Figure S2. Lactate decreases, whereas LDHA inhibition increases, ferroptosis sensitivity in GSCs.**
**(A, B)** Cell viability of PN GSCs MGG8 **(A)** and PN35 **(B)** treated with Erastin or RSL3 with or without lactate supplementation. Cells were pretreated with lactate (12 mM, 24 h) and then treated with Erastin (5 μM, 6 h) or RSL3 (0.1 μM, 24 h). **(C–E)** Representative C11-BODIPY flow cytometry histograms **(C)** and quantification of lipid ROS levels in MGG8 **(D)** and PN35 **(E)** cells under the indicated treatments. **(F, G)** Cell viability of MES GSCs WL1 **(F)** and 2907 **(G)** treated with Erastin or RSL3 with or without stiripentol-mediated LDHA inhibition. Cells were pretreated with stiripentol (0.5 mM, 24 h) and then treated with Erastin (5 μM, 6 h) or RSL3 (0.1 μM, 24 h). **(H–J)** Representative C11-BODIPY flow cytometry histograms **(H)** and quantification of lipid ROS levels in WL1 **(I)** and 2907 **(J)** cells under the indicated treatments. Lipid ROS was measured using BODIPY 581/591 C11 (1.5 μM, 30 min). Data are shown as mean ± SEM from n = 3 independent biological experiments. Statistical significance was determined by two-way ANOVA followed by Tukey’s multiple-comparison test. *P < 0.05, **P < 0.01, ***P < 0.001.

**Figure S3. MES-associated transcription factors promote LDHA transcription in MES GSCs.**

**(A–C)** qRT-PCR analysis **(A)**, immunoblot analysis **(B)**, and quantification **(C)** of C/EBPβ expression in WL1 and 2907 MES GSCs transfected with control siRNA or two independent siRNAs targeting C/EBPβ. **(D–F)** qRT-PCR analysis **(D)**, immunoblot analysis **(E)**, and quantification **(F)** of STAT3 expression in WL1 and 2907 cells transfected with control siRNA or two independent siRNAs targeting STAT3. **(G–I)** qRT-PCR analysis **(G)**, immunoblot analysis **(H)**, and quantification **(I)** of TAZ expression in WL1 and 2907 cells transfected with control siRNA or two independent siRNAs targeting TAZ. **(J–L)** qRT-PCR analysis **(J)** of LDHA mRNA levels, immunoblot analysis **(K)** and quantification **(L)** of LDHA protein expression in WL1 and 2907 cells after knockdown of C/EBPβ, STAT3, or TAZ. **(M)** Measurement of intracellular lactate levels in WL1 and 2907 cells after knockdown of C/EBPβ, STAT3, or TAZ. **(N)** Schematic representation of the LDHA promoter showing predicted C/EBPβ, STAT3, and TEAD/TAZ-associated binding motifs and the ChIP-qPCR primer regions P1–P4. **(O, P)** ChIP-qPCR analysis of C/EBPβ, p-STAT3, and TEAD/TAZ enrichment at the LDHA promoter in 2907 **(O)** and WL1 **(P)** cells. IgG was used as the negative control. P1, P2, and P3 correspond to predicted C/EBPβ, STAT3, and TEAD/TAZ-associated motif regions, respectively, whereas P4 was used as an optional control region. Data are presented as mean ± SEM from n = 3 independent biological experiments. Statistical significance was determined by unpaired two-tailed Student’s t-test or one-way ANOVA followed by Tukey’s multiple-comparison test, as appropriate. *P < 0.05, **P < 0.01, ***P < 0.001; ns, not significant.

**Figure S4. MBOAT2 suppresses radiation-induced ferroptosis through PE-MUFA remodeling in MES GSCs.**

**(A)** Representative C11-BODIPY flow cytometry histograms showing lipid ROS levels in WL1 and 2907 MES GSCs with MBOAT2 overexpression or MBOAT2 knockdown under basal or irradiated conditions. **(B, C)** Quantification of lipid ROS levels **(B)** and γ-H2AX-positive cells **(C)** in WL1 and 2907 cells with MBOAT2 overexpression or knockdown, with or without irradiation. MBOAT2 overexpression reduced radiation-induced lipid ROS accumulation and γ-H2AX foci formation, whereas MBOAT2 knockdown enhanced these effects. **(D)** Representative γ-H2AX immunofluorescence images corresponding to **(C)**. γ-H2AX-positive cells were defined as cells containing more than 10 nuclear γ-H2AX foci. Scale bar, 50 μm. **(E)** Representative C11-BODIPY flow cytometry histograms showing the effects of PE-OA 18:1 or PE-FA 20:4 supplementation on lipid ROS accumulation in control or MBOAT2-depleted WL1 and 2907 cells under basal or irradiated conditions. **(F, G)** Quantification of lipid ROS levels **(F)** and γ-H2AX-positive cells **(G)** in MBOAT2-depleted WL1 and 2907 cells supplemented with PE-OA 18:1 or PE-FA 20:4, with or without irradiation. PE-OA 18:1 partially rescued the increase in lipid ROS and γ-H2AX-positive cells caused by MBOAT2 depletion, whereas PE-FA 20:4 showed limited rescue. **(H)** Representative γ-H2AX immunofluorescence images corresponding to **(G)**. Scale bar, 50 μm. **(I)** Representative C11-BODIPY flow cytometry histograms showing lipid ROS levels in irradiated MBOAT2-depleted WL1 and 2907 cells treated with Fer-1, PE-OA 18:1, or their combination. **(J, K)** Quantification of lipid ROS levels **(J)** and γ-H2AX-positive cells **(K)** in irradiated MBOAT2-depleted WL1 and 2907 cells treated with Fer-1, PE-OA 18:1, or their combination. Fer-1 and PE-OA 18:1 reduced the ferroptosis-associated and DNA damage phenotypes induced by MBOAT2 depletion under irradiation. **(L)** Representative γ-H2AX immunofluorescence images corresponding to **(K)**. Scale bar, 50 μm. Cells were irradiated with 4 Gy where indicated. γ-H2AX foci were analyzed at 12 h post-irradiation. Lipid ROS was detected using BODIPY 581/591 C11 (1.5 μM, 30 min). PE-OA 18:1 and PE-FA 20:4 were used at 20 μM for 12 h before irradiation or lipid ROS analysis. Fer-1 was used at 1 μM for 24 h. Data are presented as mean ± SEM from n = 3 independent biological experiments. Statistical significance was determined by one-way ANOVA followed by Tukey’s multiple-comparison test. *P < 0.05, **P < 0.01, ***P < 0.001.

**Figure S5. Expression validation of MBOAT2-WT and MBOAT2-H373A in GSCs.** **(A-B)** Immunoblot analysis of MBOAT2 expression in WL1 **(A)** and 2907 **(B)** GSCs transduced with vector control, MBOAT2-WT, or the catalytically impaired MBOAT2-H373A mutant.

**Figure S6. Exogenous lactate partially delays MBOAT2 degradation in LDHA-depleted GSCs.**
**(A, C)** CHX chase assay showing MBOAT2 protein stability in lactate-treated control and LDHA-depleted WL1 **(A)** and 2907 **(C)** GSCs. Cells were supplemented with lactate (12 mM, 24 h) and then treated with CHX (100 μg/mL) for the indicated time points. GAPDH was used as the loading control. **(B, D)** Quantification of MBOAT2 protein levels in WL1 **(B)** and 2907 **(D)** cells. MBOAT2 was normalized to GAPDH and expressed relative to the 0 h time point. Data are shown as mean ± SEM from n = 3 independent biological experiments. *P < 0.05, **P < 0.01.

**Figure S7. Histone lactylation landscape and p300 expression in MES and PN GSCs.**

**(A, B)** Immunoblot analysis **(A)** and quantification **(B)** of histone-associated pan-Kla levels in MES and PN GSCs. Pan-Kla signals in the histone region were normalized to Histone H3. **(C, D)** Immunoblot analysis **(C)** and quantification **(D)** of site-specific histone lactylation marks in MES and PN GSCs. H3 lactylation marks were normalized to total Histone H3, and H4 lactylation marks were normalized to total Histone H4. **(E, F)** Immunoblot analysis **(E)** and quantification **(F)** of p300 protein expression in MES and PN GSCs. p300 was normalized to GAPDH. **(G)** Quantification of p300 knockdown efficiency in WL1 and 2907 cells. Data are presented as mean ± SEM from n = 3 independent biological experiments. *P < 0.05, **P < 0.01; ns, not significant.

**Figure S8. USP30 directly interacts with MBOAT2 and stabilizes MBOAT2 by limiting Lys48 ubiquitination.**
**(A)** Reciprocal endogenous Co-IP analysis of USP30 and MBOAT2 interaction in WL1 and 2907 GSCs. MBOAT2 or USP30 was immunoprecipitated as indicated, and the reciprocal interacting protein was detected by immunoblotting. Normal IgG was used as a negative control. **(B)** GST pull-down assay showing direct interaction between GST-MBOAT2 and Flag-USP30. GST alone was used as the negative control. GST and GST-MBOAT2 proteins were visualized by anti-GST staining. **(C)** Immunoblot analysis of MBOAT2 expression in WL1 and 2907 GSCs transfected with increasing amounts of Flag-USP30 wild-type or the catalytically inactive USP30-C77A mutant. **(D)** MBOAT2 ubiquitination assay in WL1 and 2907 GSCs expressing Myc-MBOAT2 together with vector, Flag-USP30 wild-type, or Flag-USP30-C77A. MBOAT2 was immunoprecipitated with anti-Myc antibody, and ubiquitinated MBOAT2 was detected by immunoblotting with anti-ubiquitin antibody. Quantification of ubiquitinated MBOAT2 is shown on the right. **(E)** Ubiquitination analysis of wild-type MBOAT2 and candidate lysine-to-arginine mutants in WL1 and 2907 cells. Cells were co-transfected with Myc-tagged MBOAT2 constructs, HA-ubiquitin, and Flag-USP30 as indicated. MBOAT2 was immunoprecipitated with anti-Myc antibody, and ubiquitinated MBOAT2 was detected by immunoblotting with anti-HA antibody. **(F)** Immunoblot analysis of wild-type MBOAT2 and MBOAT2-Lys48R protein levels in WL1 and 2907 cells with or without USP30 knockdown. Data are presented as mean ± SEM from n = 3 independent biological experiments where applicable. **P < 0.01, ***P < 0.001.

**Figure S9. Dose and treatment-schedule optimization of oxamate plus USP30-IN-20 combined with radiotherapy in intracranial GSC xenografts.**

**(A)** Representative IHC staining for 4-HNE and Ki67 and H&E staining of brain sections from mice receiving low-, intermediate-, or high-dose combinations of oxamate and USP30-IN-20 together with local cranial irradiation. 4-HNE was used as a lipid peroxidation marker, and Ki67 was used as a proliferation marker. **(B)** Quantification of 4-HNE and Ki67 staining shown in **(A)**. IHC staining was quantified based on staining intensity and the proportion of positive tumor cells. **(C)** Kaplan–Meier survival analysis of mice receiving low-, intermediate-, or high-dose combination treatment. **(D)** Representative IHC staining for 4-HNE and Ki67 and H&E staining of brain sections from mice treated with different administration schedules of oxamate and USP30-IN-20 combined with irradiation. In the synchronous schedule, oxamate and USP30-IN-20 were administered together during the irradiation period. In the sequential schedule, mice were pretreated with oxamate before USP30-IN-20 administration and irradiation. **(E)** Quantification of 4-HNE and Ki67 staining shown in **(D)**. **(F)** Kaplan–Meier survival analysis of mice treated with the indicated administration schedules. For in vivo treatment, oxamate was administered intraperitoneally, USP30-IN-20 was administered by oral gavage, and local cranial irradiation was delivered as 2.5 Gy per fraction for four consecutive days. The optimized regimen selected for subsequent experiments was oxamate (300 mg/kg, intraperitoneally, once daily) and USP30-IN-20 (10 mg/kg, oral gavage, once daily). Data are shown as mean ± SEM. n = 5 male nude mice per group. Statistical significance was determined by one-way ANOVA or log-rank test, as appropriate. *P < 0.05, **P < 0.01, ***P < 0.001.

**Supplementary Table 1**

| **Sequences for shRNAs** | |
| --- | --- |
| shp300-1 | 5′-CCAATGGTGGTGATATTAATC -3′ |
| shp300-2 | 5′-GGATTATGACTTGTGTATCAC-3′ |
| shLDHA-1 | 5′-CCAAAGATTGTCTCTGGCAAA-3′ |
| shLDHA-2 | 5′-ATCCAGTGGATATCTTGACCTA-3′ |
| shUSP30-1 | 5′-CCTGGATCATCTACGACTACAA-3′ |
| shUSP30-2 | 5′-GATCAAGGACTTCAGCAAGAA-3′ |
| shMBOAT2-1 | 5′-GCTCTTACAAAGACTACATTA-3′ |
| shMBOAT2-2 | 5′-GCAAGTCTTCTTCTGCTATTA-3′ |

**Supplementary Table 2**

| **Primer sequences** | |
| --- | --- |
| LDHA | forward: 5′- AGCTGTTCCACTTAAGGCCC-3′  reverse: 5′- AATGAGATCCGGAATCGGCG-3′ |
| Glut1 | forward: 5′-GGCCAAGAGTGTGCTAAAGAA-3′  reverse: 5′- ACAGCGTTGATGCCAGACAG-3′ |
| Gys1 | forward: 5′- CACAGAACGGTTGTCGGACTTG-3′  reverse: 5′- AGGTGAAGTGGTCTGGAAAGGC-3′ |
| Agl | forward: 5′-CAGGATGCTGCTGAGGTTCT-3′ |
|  | reverse: 5′- TGGTGGTGATGTTGAGGTTG-3′ |
| Mct1 | forward: 5′- GGTGGAGGTCCTATCAGCAGT-3′  reverse: 5′- CAGAAAGAAGCTGCAATCAAGC-3′ |
| Panx1 | forward: 5′- GGTCTACCTCTGCTGCCTCATCT-3′  reverse: 5′- CCAGCAGTATGAATCCACAAAGG-3′ |
| MBOAT2 | forward: 5′- CTCGCTGGGACTTAATTTCCAA-3′  reverse: 5′- GGTTCGTTCATAACACACCCTT-3′ |
| EP300 / p300 | forward: 5′- GAGAGCTGGAAGATGAGGGA -3'  reverse: 5'- AGGAACAGGAGGAGGAGTGA -3' |
| USP30 | forward: 5′- GACCTGGCGTGAGTGTTCT-3'  reverse: 5′- TGTCCAGGGAAGGGAGTGTA-3' |
| CEBPB / C/EBPβ | forward: 5′-AACTCTCTGCTTCTCCCTCTG-3′  reverse: 5′-AAGCCCGTAGGAACATCTTTAA-3′ |
| STAT3 | forward: 5′-CAGCAGCTTGACACACGGTA-3′  reverse: 5′-AAACACCAAAGTGGCATGTGA-3′ |
| WWTR1 / TAZ | forward: 5′-AGTACCCTGAGCCAGCAGAA-3′  reverse: 5′-GATTCTCTGAAGCCGCAGTT-3′ |
| GAPDH | forward: 5′- GTCTCCTCTGACTTCAACAGCG-3′  reverse: 5′-ACCACCCTGTTGCTGTAGCCAA-3′ |

**Supplementary Table 3 Antibodies and reagents used in this study.**

The antibodies, chemical inhibitors, fluorescent probes, and other reagents used for immunoblotting, immunofluorescence, immunohistochemistry, flow cytometry, and functional assays are listed with their corresponding suppliers and catalog numbers.

| Antibodies or reagents | Source | Identifier |
| --- | --- | --- |
| RSL3 | Selleck Chemicals | S8155 |
| Ferrostatin-1 | Selleck Chemicals | S7243 |
| Z-VAD-FMK | Selleck Chemicals | S7023 |
| GPX4 | Abcam | ab125066 |
| SCD1 | Abcam | ab39969 |
| TAZ | Abcam | ab84927 |
| C/EBPβ | Abcam | ab32358 |
| Histone H4 | Abcam | ab177840 |
| OLIG2 | Abcam | ab81093 |
| SOX2 | Abcam | ab79351 |
| LDHB | Abcam | ab53292 |
| Caspase-3 | Cell Signaling Technology | 9662 |
| CD44 | Cell Signaling Technology | 3570 |
| p-STAT3 | Cell Signaling Technology | 9145 |
| STAT3 | Cell Signaling Technology | 9139 |
| His-Tag | Cell Signaling Technology | 2366 |
| HA-Tag | Cell Signaling Technology | 3724S |
| mouse IgG | Cell Signaling Technology | 5415 |
| rabbit IgG | Cell Signaling Technology | 3900 |
| H2AX | Cell Signaling Technology | 7631 |
| H2AX (Ser139) | Cell Signaling Technology | 9718 |
| Histone H3 | Cell Signaling Technology | 4620 |
| p300 | Cell Signaling Technology | 86377 |
| Ki67 | Santa Cruz Biotechnology | sc-101861 |
| FSP1 | Santa Cruz Biotechnology | sc-377120 |
| USP30 | Santa Cruz Biotechnology | sc-515235 |
| GAPDH | Invitrogen | MA5-15738 |
| BODIPY 581/591 C11 | Invitrogen | D3861 |
| Erastin | MCE | #HY-15763 |
| Necrostatin-1 | MCE | #HY-15760 |
| Stiripentol | MCE | #[HY-103392](https://www.medchemexpress.cn/Stiripentol.html) |
| [USP30-IN-20](https://www.medchemexpress.cn/usp30-in-20.html) | MCE | #[HY-175826](https://www.medchemexpress.cn/usp30-in-20.html) |
| Oxamate | MCE | #[HY-W013032A](https://www.medchemexpress.cn/oxamic-acid-sodium.html) |
| Bafilomycin A1 | MCE | [HY-100558](https://www.medchemexpress.cn/Bafilomycin_A1.html) |
| MG132 | MCE | [HY-13259](https://www.medchemexpress.cn/MG-132.html) |
| C646 | MCE | [HY-13823](https://www.medchemexpress.cn/C646.html) |
| A-485 | MCE | HY-107455 |
| Flag-Tag | Sigma-Aldrich | catalog F3165 |
| MCT1 | Proteintech | 20139-1-AP |
| LDHA | Proteintech | 19987-1-AP |
| pan-Kla | PTM BIO | PTM-1401 |
| pan-Kac | PTM BIO | PTM-101 |
| Lactyl-Histone H3 (Lys9) | PTM BIO | PTM-1419RM |
| Lactyl-Histone H3 (Lys23) | PTM BIO | PTM-1413RM |
| Lactyl-Histone H3 (Lys27) | PTM BIO | PTM-1428 |
| Lactyl-Histone H3 (Lys56) | PTM BIO | PTM-1421RM |
| Lactyl-Histone H3 (Lys18) | PTM BIO | PTM-1406RM |
| Lactyl-Histone H3 (Lys14) | PTM BIO | PTM-1414RM |
| Lactyl-Histone H4 (Lys8) | PTM BIO | PTM-1415RM |
| Lactyl-Histone H4 (Lys5) | PTM BIO | PTM-1407RM |
| Lactyl-Histone H4 (Lys12) | PTM BIO | PTM-1411RM |
| Lactyl-Histone H4 (Lys16) | PTM BIO | PTM-1417RM |
| MBOAT2 | Novus Biologicals | C#NBP182236 |

**Supplementary Table 4**

| **Sequences for siRNAs** | | |  |
| --- | --- | --- | --- |
|  | Sense / target sequence | Antisense sequence | |
| siCEBPB-1 | 5′-CGACUUCCUCUCCGA  CCUCUU-3′ | 5′-AAGAGGUCGGAGAGGAA  GUCG-3′ | |
| siCEBPB-2 | 5′-GAAGACCGUGGACAA  GCAC-3′ | 5′-GUGCUUGUCCACGGUCU  UC-3′ | |
| siSTAT3-1 | 5′-CGUCAUUAGCAGAAUC  UCAdTdT-3′ | | 5′-UGAGAUUCUGCUAAUGAC  GdTdT-3′ |
| siSTAT3-2 | 5′-GCAGCAGCUGAACAAC  AUGU-3′ | | 5′-ACAUGUUGUUCAGCUG  CUGC-3′ |
| siTAZ-1 | 5′-GGGAAAGUGAACAUG  AGUUdTdT-3′ | | 5′-AACUCAUGUUCACUUU  CCCdTdT-3′ |
| siTAZ-2 | 5′-UGACGUCCUUCCUAA  CAGUdTdT-3′ | | 5′-ACUGUUAGGAAGGACGU  CAdTdT-3′ |

**Supplementary Table 5**

| **ChIP-qPCR primer sequences** | | |  |
| --- | --- | --- | --- |
| Target region | Target region | Target region | |
| LDHA promoter P1 | 5′-AGGCTGAGGCAGGAGAATCA-3′ | 5′-AGGCTGAGGCAGGAGAATCA-3′ | |
| LDHA promoter P2 | 5′-CCTGCTGTGACCTGAGATGA-3′ | 5′-CCTGCTGTGACCTGAGATGA-3′ | |
| LDHA promoter P3 | 5′-GAGGCGGAGAGGAGAGAGAA-3′ | | 5′-GAGGCGGAGAGGAGAGAGAA-3′ |
| LDHA promoter P4 | 5′-CGGAGGAGGAGGAGGAGATA-3′ | | 5′-CGGAGGAGGAGGAGGAGATA-3′ |
| USP30 promoter | 5′-GCTGAGGAGGAGAGGAGGAA-3′ | | 5′-GCTGAGGAGGAGAGGAGGAA-3′ |
